# Geometrization for Energy Levels of Isotropic Hyperfine Hamiltonian Block and Related Central Spin Problems for an Arbitrarily Complex Set of Spin-1/2 Nuclei

**DOI:** 10.3390/ijms232315199

**Published:** 2022-12-02

**Authors:** Dmitri V. Stass

**Affiliations:** 1 Voevodsky Institute of Chemical Kinetics and Combustion, 630090 Novosibirsk, Russia; stass@ns.kinetics.nsc.ru; 2 International Tomography Center, 630090 Novosibirsk, Russia

**Keywords:** hyperfine interaction, central spin Hamiltonian, nuclear hyperpolarization, eigenvalue, matrix factorization, geometric visualization, discrete Fourier transform, Laplacian matrix, weighted graph

## Abstract

Description of interacting spin systems relies on understanding the spectral properties of the corresponding spin Hamiltonians. However, the eigenvalue problems arising here lead to algebraic problems too complex to be analytically tractable. This is already the case for the simplest nontrivial $\left(K_{\max}-1\right)$ block for an isotropic hyperfine Hamiltonian for a radical with spin-12 nuclei, where n nuclei produce an *n*-th order algebraic equation with *n* independent parameters. Systems described by such blocks are now physically realizable, e.g., as radicals or radical pairs with polarized nuclear spins, appear as closed subensembles in more general radical settings, and have numerous counterparts in related central spin problems. We provide a simple geometrization of energy levels in this case: given n spin-12 nuclei with arbitrary positive couplings $a_{i},$ take an *n*-dimensional hyper-ellipsoid with semiaxes $\sqrt{a_{i}},$ stretch it by a factor of $\sqrt{n+1}$ along the spatial diagonal (1, 1, …, 1), read off the semiaxes of thus produced new hyper-ellipsoid $q_{i},$ augment the set $\left\{q_{i}\right\}$ with $q_{0}=0,$ and obtain the sought n+1 energies as $E_{k}=-\frac{1}{2}q_{k}^{2}+\frac{1}{4}\sum_{i}a_{i}.$ This procedure provides a way of seeing things that can only be solved numerically, giving a useful tool to gain insights that complement the numeric simulations usually inevitable here, and shows an intriguing connection to discrete Fourier transform and spectral properties of standard graphs.

## 1. Introduction

Solving a Hamiltonian for a physical system with n free parameters naturally leads to the need to solve an algebraic problem with n free parameters, e.g., derive and solve a secular equation to find the energies of eigenstates as the roots of an at least *n*-th order polynomial. However, unless the situation is very symmetric, allows a perturbative treatment, or can be physically factored into lower-dimensional subtasks, anything beyond a cubic equation is an analytical dead-end, and one is faced with the choice of either resorting to simple models with no more than three parameters, or having to go numeric.

This work focuses on the particular problem of describing the energy levels of the spin system of an organic radical with electronic spin-12 interacting with spin-12 nuclei via isotropic hyperfine interaction described by Hamiltonian

(1) $$\hat{H}=\sum_{i=1}^{n}a_{i}\vec{S}\cdot {\vec{I}}_{i}.$$

This is a representative case of the central spin problem in science, one selected spin interacting with multiple non-interacting spins of a different kind, or a localized spin coupled to a “spin bath,” and parallels can be found in many problems of condensed matter and chemical physics and quantum information processing. Some examples apart from the already quoted organic radical or radical pairs include electrons in quantum dots [1] and double dots [2], Rydberg electrons in cold bosonic clouds [3], fermionic condensates [4], optically addressable paramagnetic defects in semiconductors [5], Gaudin magnets [6], general quantum impurity problems [7], periodically driven many-body quantum systems [8], dynamic polarization protocols [9], general electron spin hyperfine coupled to a large number of nuclear spins [10], quantum memory for qubit states [11], mixed electron-nuclear spin qubits [12], spin qubit initialization and error-correction protocols [13], control of spin bath via central spin [14], or superconductor quantum computing [15]. For convenience the language of radical/radical pair will be used for derivation, but the methods and results can be transferred to other similar problems in adjacent fields. Some analytic models and asymptotic estimates have been developed for description of such spin systems, in particular for very large spin baths with statistical distribution of couplings, as can be found in the cited references. However, systems with a finite number of spins and arbitrary couplings still mostly require direct numerical simulations, and interpretation of their results is an additional, often quite substantial, problem.

Although the task of describing the spin system of an organic radical with isotropic hyperfine interaction may look simple for a physical problem, and in a strong applied magnetic field general perturbative solutions have long been available, the situation for weak or zero applied field is drastically more difficult. After the simple cases have been covered in early works, as summarized, e.g., in monograph [16], further advances have been rather scarce and included just the simplest systems with only few nuclei [17], special classes of systems having all equivalent nuclei [18,19] or two groups of equivalent nuclei [20], and approximations such as semiclassical [21] and one dominant coupling [22]. The algebraic complexity in any system beyond the listed simplest cases forces to turn to numeric simulations [23,24,25]. 

The area of radicals and radical pairs in close to zero magnetic fields used to be a specialty and rather narrow subfield of magnetic resonance and spin chemistry, limited to topics such as zero-field line in magnetic field effect on radical pair recombination [26,27,28,29,30] going back to Hanle effect in gas-phase fluorescence [31], or putative mechanisms of animal magnetoreception [32,33,34]. However, recent years have seen a resurgence of general interest to coupled spin systems in weak and zero magnetic field due to advances in NMR relying on strongly J-coupled nuclear spin systems described by nearly identical Hamiltonians, such as NMR of hyperpolarized nuclei via *Para*-hydrogen Induced Polarization [35,36,37] and Zero and Ultra Low Field (ZULF) NMR [38,39,40,41,42], where polarization transfer is effective in a relatively narrow range of fields in the region of avoided crossing of spin system energy levels [43,44], quantum magnetometry in low field [45], and revisiting magnetic field effects in covalently coupled [46,47,48] or supramolecularly assembled [49] donor-acceptor systems with spin-dependent charge separation and recombination as possible artificial photosynthesis mimics. Such spin systems are usually again treated exactly in simple few-nuclei or highly symmetric cases that allow this [50,51,52], or are approached numerically for multinuclear situations [53].

This paper pursues a different approach to the problem of algebraic complexity in describing spin system of a radical in zero field, explored earlier in [54]. Rather than trying to completely solve a model for such a system simple and trivial enough to allow this, we consider a system of an arbitrary generality, in which a physically reasonable part representative of its behavior can be singled out that still allows a useful analytical insight. The complexity then forces to search for trends and generalizations that could have been overlooked in a simpler system or a numerical simulation. In particular, that paper discussed in details that representing matrix for the hyperfine Hamiltonian in zero field (1), which has dimensions $2^{n+1}\times 2^{n+1},$ is block-diagonal in the natural Zeeman basis, the blocks are indexed by the conserved total spin projection, and already the first nontrivial “penultimate” sub-block with total electron and nuclei spin projection one step lower than maximum

(2) $$K=S_{z}+\sum_{i}I_{iz}=K_{\max}-1=(n-1)/2,$$

which has dimensions (n+1)×(n+1), is a separate entity of key importance for describing recombining spin-correlated radical pairs that feature one or both radicals with an arbitrary set of n spin-12 nuclei. 

This importance has two aspects. The first one relates to a very general situation of photo- or radiation-induced radical ion pairs in low-viscosity liquids that are formed in singlet initial spin state of the two unpaired electrons and are observed via recombination also from singlet collective spin state [55,56,57]. Such pairs are reasonably well described by considering only dynamic spin evolution driven by intra-radical isotropic hyperfine interactions, omitting inter-radical spin-spin interactions and spin relaxation, and therefore are described by a sum of two independent Hamiltonians of the type (1), which conserves total spin projection of the pair. All such systems include a subensemble of pairs starting from initial singlet electron spin state with all nuclear spins aligned in the same direction. Such a subensemble is present for a radical pair of arbitrary hyperfine complexity, and under stated assumptions its evolution is independent of other subensembles that must have a different total spin projection of the pair. Therefore, its contribution to recombination probability is additive. Furthermore, this evolution can be described using only the state subspaces of the individual radicals with either maximum total (electron plus nuclei) spin projection, or total projection one less than maximum (2). Thus, to describe this subensemble it suffices to solve the “penultimate” block of the hyperfine Hamiltonian for a radical, as the state with the maximum total spin projection is automatically an eigenstate and needs no efforts. Although the statistical weight of the “all nuclear spins up” subensemble of radical pairs rapidly decreases with increasing the number of nuclei, its additive contribution to any experimental observable is always present, and as discussed in [54] its behavior is in a sense typical for more complex subensembles with lower total spin projection. In particular, a perturbative treatment of applied weak magnetic field is possible in the most general case and gives such insights as an expected linear skew in magnetic field dependence of recombination probability upon passing through zero field if the weights of “all nuclear spins up” and “all nuclear spins down” subensembles are not equal, i.e., nuclear spins are polarized. Such a skew indeed was implicitly present in model calculations [58], although it has yet to be observed in experiment. This would be an interesting possibility of introducing anisotropy (via polarized nuclear spins, i.e., via the initial state) in an otherwise intrinsically isotropic spin system.

The second aspect is related to the abovementioned option of having radicals with initially polarized nuclear spins. One of the crucial paradigm shifts in NMR over the last two decades is a realization that non-Boltzmannian nuclear population (hyperpolarization) is possible and can be created by several means. The already implemented approaches include dynamic nuclear polarization DNP [59,60], chemically induced dynamic nuclear polarization CIDNP [61,62], optical nuclear polarization ONP [63], optical pumping of noble gases [64] or semiconductors [65], *para*-hydrogen induced polarization (PHIP) [66] and Signal Amplification By Reversible Exchange (SABRE) [67,68]. Achieving an orders of magnitude boost in NMR sensitivity, these methods are capable of producing diamagnetic systems with nuclear polarizations of percents to dozens of percent persisting for times of the order of nuclear relaxation times, seconds for hydrogens to minutes for nuclei with lower magnetogyric ratios, or maintained indefinitely in case of continuous generation. Much longer-lived collective symmetry-protected nuclear spin states can be created [69,70,71,72,73], and can be used as reservoirs for accumulating spin polarization that can then be released into the spin system. For the simplest possible systems of two spin-12 nuclei the symmetry-protected state is usually the non-magnetic singlet state, with net magnetization carried by triplet states, and methods are now available for efficient conversion of triplet states to singlet and back (magnetization-to-singlet, M2S, and singlet-to-magnetization, S2M, conversion) [74,75,76,77,78]. Larger systems of up to 4 spins have been successfully polarized [79,80]. While hyperpolarization of organic molecules was initially mostly confined to hydrogens due to availability of a convenient source of hyperpolarized hydrogens in the form of molecular *para*-hydrogen, the more recent trend is polarization transfer to heteronuclei-like ^13^C and ^15^N [81,82,83,84,85,86]. Furthermore, the most efficient redistribution of hyperpolarization over the system of coupled nuclei occurs in the conditions of strong J-coupling, when scalar coupling of spins is larger than or comparable to the difference of their Zeeman energies. For homonuclear systems, like J-coupled hydrogens, this usually happens in milliTesla fields, while efficient coupling of heteronuclei like ^13^C-^1^H pairs requires microTesla to nanoTesla fields due to vastly different magnetogyric ratios, which was one of the main drivers for developing ZULF NMR. Thus, the new reality in the NMR community is availability of hyperpolarized diamagnetic systems in close to zero fields with amounts, lifetimes, and degrees of polarization already sufficient for in-vivo use as MRI agents. But then the new experimental reality for the radical/radical pair community is availability of hyperpolarized starting systems to generate radicals from, with substantial polarization at ^13^C and ^15^N spin-12 nuclei that often show higher hyperfine coupling constants than protons and will thus dominate the hyperfine structure of the radical. To the best of our knowledge, no such experiments on radicals or radical pairs with initially hyperpolarized nuclei have been reported so far, but this is certainly an emerging experimental possibility.

Thus, the penultimate block of the hyperfine Hamiltonian is indeed a “physically reasonable part” of the problem that may be practically realizable by itself, provides useful insights on more complex parts of a more general problem, and still allows fairly deep analysis without resorting to numeric simulations. The paper [54] had derived the secular equation for this block and certain spectral bounds for its eigenvalues. Still for n nuclei the problem has n hyperfine coupling constants $a_{i}$ as free parameters, and denies a general analytic solution, although the eigenvalues can certainly be found numerically by diagonalizing the initial matrix.

It often happens that things that are easy to imagine or draw free-hand are difficult to describe analytically. The prime example of this is computer graphics, which has long become a research field in applied mathematics in itself. Occasionally this complexity may work the other way round, and things that are difficult to treat analytically may happen to have an intuitively natural and appealing visualization. This work will provide such a visualization for the energies of the penultimate Hamiltonian block described above: given n spin-12 nuclei with arbitrary positive couplings $a_{i},$ take an *n*-dimensional hyper-ellipsoid centered at the origin of the reference frame and having semiaxes $\sqrt{a_{i}}$ aligned with the *n* Cartesian axes, stretch it by a factor of $\sqrt{n+1}$ along the spatial diagonal (1,1,…,1), and the semiaxes of the obtained new hyper-ellipsoid $q_{i},$ augmented with one special value $q_{0}=0,$ produce the n+1 energy levels as $E_{k}=-\frac{1}{2}q_{k}^{2}+\frac{1}{4}\sum_{i}a_{i}.$ While such a procedure cannot yield analytic expressions for the eigenvalues and these would be computed numerically anyway, as the roots of the corresponding high-order polynomial cannot be found analytically for algebraic reasons, it is believed that such thinking of Hamiltonians in terms of geometric shapes can be useful in supporting the intuition and providing insights to complement the usually inevitable numeric calculations.

This work does not compete with the existing spin dynamics simulation software [87,88,89,90,91,92,93,94,95,96,97] and pursues a different goal. Its raison d’etre is not how to compute, but rather how to not compute and still obtain useful insights about spin dynamics. The purpose is to obtain a picture of the core, spectral, properties of the central spin Hamiltonian that will eventually show up in any simulation, rather than to generate a visual representation of spin dynamics. The notion of (hyper-)ellipsoids central to this work may also sound misleadingly familiar. Any purely dynamic spin system completely described by Hamiltonian can in one sense or another be represented by some ellipsoids, as there is a set of purely real eigenvalues that can be all made positive by a suitable shift of energy origin, and these are algebraically equivalent to ellipsoids. On the other hand, ordinary 3D-ellipsoids are very familiar for magnetic resonance community, as they are used to represent, e.g., chemical shielding and shift tensors, dipole–dipole and quadrupolar couplings, and atomic anisotropic displacement parameters (thermal ellipsoids) derived from NMR crystallography in NMR [98], or anisotropic hfi tensors and g-tensors in EPR [99]. However, the point of this work is a very special arbitrary-dimensional ellipsoid with direct connection to initial parameters $a_{i}$ and a startlingly simple, universal visualization that does not require any computation at all for an arbitrary complex system.

All the derivations in this work rely on basic complex/matrix algebra covered in university textbooks, e.g., [100,101,102] and probably do not require specific references, several more specific simple facts that are used are reproduced just inline instead of citing them. Connections to more remote areas that are not further explored here, such as Laplacian matrices for graphs and Discrete Fourier Transform, will only be indicated at suitable places. The usual conventions of $\mathrm{diag}\left\{a_{1},\;a_{2},\;\ldots ,\;a_{n}\right\},$ $\hat{1},$ |M|, ${\left(\ldots \right)}^{T}$ and ${\left(\ldots \right)}^{+}$ will be used to denote a diagonal matrix with the only nonzero elements being $a_{i}$ at diagonal, identity matrix, determinant of matrix, matrix transpose and Hermitian conjugate, ket notation $|\phi_{k}\rangle$ will also be used for vectors.

The paper is organized as follows. First we briefly introduce the used notation and summarize the necessary results from [54] that will be used as the starting point for the derivations of this work, and formulate the problem of obtaining the eigenvalues (roots) of a certain matrix derived from Hamiltonian block. Then a zero-splitting transform is built in two flavors, an explicitly complex unitary one and a real orthogonal one, which both remove the present zero eigenvalue from the matrix/secular equation and produce a more convenient positive definite matrix, either complex Hermitian or real symmetric, respectively, inheriting all the non-zero roots. The real zero-split matrix is then used to introduce the circulant form and as a bridge to the reference case of equivalent nuclei. The scaling and diagonalizing transforms are built for the complex zero-split matrix M that together produce a diagonal matrix $\mathrm{diag}\{a_{1},\;\ldots ,\;a_{n}\}$ from which M can be reconstructed as a simple factorization, the inverse matrix for M is constructed, and matrix M is used to treat the loss of equivalence as a perturbation. In the last sections the stretching transform outlined above is built using *n*-dimensional hyper-ellipsoid as a geometric image of the positive definite matrix, and the circle is completed by showing that the original characteristic polynomial can be obtained by variation of a functional produced by the stretching. The concluding section summarizes the essential result and briefly discusses its connection to solving Hamiltonians for systems of interacting spins.

## 2. Results and Discussion

### 2.1. Notation and Previous Results

This section introduces the notation and adopts the necessary results from [54] for subsequent derivations. We consider a radical having n spin-12 nuclei with arbitrary positive hyperfine coupling constants $0<a_{1}\leq a_{2}\leq \ldots \leq a_{n}$ described by isotropic hyperfine Hamiltonian (1) and its restriction to state subspace with total projection $K=S_{z}+\sum_{i}I_{iz}$ onto an arbitrarily chosen axis z that is one step less than the maximum possible value (2), spanned by n+1 product-basis functions

(3) $$|\xi_{0}\rangle =|-;+++\cdots +\rangle ;\;|\xi_{1}\rangle =|+;-++\cdots +\rangle ;\;\ldots |\xi_{n}\rangle =|+;++\cdots +-\rangle .$$

Here ± stand for projections $m=\pm \frac{1}{2},$ the first position in the notation separated with semicolon is allotted to electron spin, and the following n positions correspond to n nuclear spins, with the subscript indicating the only flipped spin in the entire set. All matrices and vectors will be written with basis ordered by increasing index. The restriction to blocks with fixed K is a natural approach for this problem, as the Hamiltonian (1), either by itself or augmented with Zeeman term, conserves the total spin projection and is thus block-diagonal with respect to K. As discussed in detail in [54], the block with $K=K_{\max}=\left(n+1\right)/2$ is trivial, the blocks with $\left|K\right|<K_{\max}-1$ are too complex for an arbitrary system, and the penultimate block $K=K_{\max}-1$ is still reasonably simple to analyze for a general system, while already rich enough to build a representative physical implementation.

The restricted Hamiltonian for this subspace is represented by an (n+1)×(n+1) real symmetric matrix

(4) $$\hat{H}=\left(\begin{array}{cccccc} -\frac{1}{4}\sum_{i}a_{i} & \frac{1}{2}a_{1} & \frac{1}{2}a_{2} & \frac{1}{2}a_{3} & \cdots & \frac{1}{2}a_{n}\\ \frac{1}{2}a_{1} & \frac{1}{4}\sum_{i}a_{i}-\frac{1}{2}a_{1} & 0 & 0 & \cdots & 0\\ \frac{1}{2}a_{2} & 0 & \frac{1}{4}\sum_{i}a_{i}-\frac{1}{2}a_{2} & 0 & \cdots & 0\\ \frac{1}{2}a_{3} & 0 & 0 & \frac{1}{4}\sum_{i}a_{i}-\frac{1}{2}a_{3} & \cdots & 0\\ \vdots & \vdots & \vdots & \vdots & \ddots & \vdots \\ \frac{1}{2}a_{n} & 0 & 0 & 0 & \cdots & \frac{1}{4}\sum_{i}a_{i}-\frac{1}{2}a_{n} \end{array}\right).$$

It is convenient to shift and scale the matrix and change its overall sign as follows:

(5) $$H\equiv \hat{\tilde{H}}=-2\left(\hat{H}-\frac{1}{4}\hat{1}\sum_{i}a_{i}\right)=\left(\begin{array}{cccccc} \sum_{i}a_{i} & -a_{1} & -a_{2} & -a_{3} & \cdots & -a_{n}\\ -a_{1} & a_{1} & 0 & 0 & \cdots & 0\\ -a_{2} & 0 & a_{2} & 0 & \cdots & 0\\ -a_{3} & 0 & 0 & a_{3} & \cdots & 0\\ \vdots & \vdots & \vdots & \vdots & \ddots & \vdots \\ -a_{n} & 0 & 0 & 0 & \cdots & a_{n} \end{array}\right).$$

All embellishments were removed from the operator symbol H for simplicity. We refer to eigenvalues $\lambda_{k}$ of (5) as “roots,” and they are related to the corresponding energies $E_{k}$ of the original Hamiltonian via an obvious relation

(6) $$E_{k}=-\frac{1}{2}\lambda_{k}+\frac{1}{4}\sum_{i}a_{i}.$$

The eigenvectors are not affected by transform (5). It can be readily seen that the vector consisting of all ones is an eigenvector of (5) with λ=0 and $E=\frac{1}{4}\sum_{i}a_{i},$ as the sum of elements in every line is equal to 0. 

**Figure 1:**
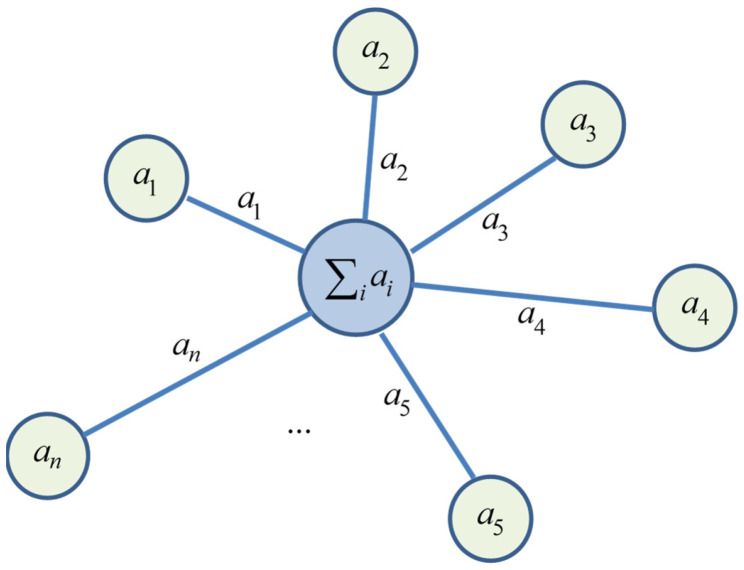
Weighted star graph representing the Hamiltonian block of this work.

The adopted form differs from the form used in [54] by overall sign change, which makes all non-zero $\lambda_{k}$ positive as is more convenient for geometrization. It may be further noted that matrix (5) looks like Laplacian, or Kirchhoff matrix for a certain graph [103], with degrees of vertices at the diagonal and minus weights of edges as off-diagonal elements. This particular matrix corresponds to a star graph shown in Figure 1, and more complex subspaces with lower |K| values will be represented by more general bipartite graphs with two classes of vertices corresponding to electron spin up or down in the relevant ket vector of the type (3), with only inter-class connections by edges with weights given by the hyperfine coupling constants for a nuclear spin flipped when flipping the electron spin to pass from a function of one class to a function of another class. The simple transform (5) is thus useful not only because it provides a convenient picture of the Hamiltonian block, but rather because it reduces it to a mathematically well-defined and thoroughly studied object [103]. For example, a Laplacian matrix for any graph has a zero eigenvalue with multiplicity equal to the number of its connected components (which is one in our case), all other eigenvalues being positive.

We also frequently meet the following symmetric combinations of the coupling constants:

(7) $$A_{0}\equiv 1;\;A_{1}=\sum_{i=1}^{n}a_{i};\;A_{2}=\sum_{\begin{array}{l} i,j=1\\ \;i<j \end{array}}^{n}a_{i}a_{j};\;A_{3}=\sum_{\begin{array}{l} i,j,k=1\\ \;i<j<k \end{array}}^{n}a_{i}a_{j}a_{k};\;\ldots ;A_{n}=\prod_{i=1}^{n}a_{i}.$$

In the adopted notation the needed results from [54] that we build upon can be summarized as follows. The secular equation to find the roots can be written in three equivalent forms:

- The starting determinant:

(8) $$0=f(\lambda )=\left|\begin{array}{cccccc} A_{1}-\lambda & -a_{1} & -a_{2} & -a_{3} & \cdots & -a_{n}\\ -a_{1} & a_{1}-\lambda & 0 & 0 & \cdots & 0\\ -a_{2} & 0 & a_{2}-\lambda & 0 & \cdots & 0\\ -a_{3} & 0 & 0 & a_{3}-\lambda & \cdots & 0\\ \vdots & \vdots & \vdots & \vdots & \ddots & \vdots \\ -a_{n} & 0 & 0 & 0 & \cdots & a_{n}-\lambda \end{array}\right|;$$

- The expanded and reduced determinant:

(9) $$\begin{array}{ll} 0=f(\lambda ) & =(A_{1}-\lambda )\prod_{i=1}^{n}(a_{i}-\lambda )-\sum_{j=1}^{n}a_{j}^{2}\prod_{\begin{array}{l} k=1\\ k\neq j \end{array}}^{n}\left(a_{k}-\lambda \right)\\ & =\left[\prod_{j=1}^{n}\left(a_{j}-\lambda \right)\right]\times \left[(A_{1}-\lambda )-\sum_{i=1}^{n}\frac{a_{i}^{2}}{a_{i}-\lambda }\right]\\ & =\left[\prod_{j=1}^{n}\left(a_{j}-\lambda \right)\right]\times \left[\lambda \left(1+\sum_{i=1}^{n}\frac{a_{i}}{a_{i}-\lambda }\right)\right]\\ & =\left[\lambda \left(1+\sum_{i=1}^{n}\frac{a_{i}}{a_{i}-\lambda }\right)\right]\;\mathrm{for}\;\mathrm{distinct}\;\mathrm{nuclei}; \end{array}$$

- The polynomial form:

(10) $$\begin{array}{l} 0=f(\lambda )=\lambda f_{n}(\lambda );\;\\ f_{n}(\lambda )={\left(-\lambda \right)}^{n}+2A_{1}{\left(-\lambda \right)}^{n-1}+3A_{2}{\left(-\lambda \right)}^{n-2}+4A_{3}{\left(-\lambda \right)}^{n-3}+\cdots +\left(n+1\right)A_{n}. \end{array}$$

Equations (8) and (10) are explicitly valid for any selection of the coupling constants $a_{i},$ while Equation (9) needs some care with equivalent nuclei ($a_{i}=a_{j}$ for some i,j), but is also valid for any set of coupling constants after all arising uncertainties have been removed. In [54] this was demonstrated in a somewhat awkward way, but, in a hindsight, it can be readily seen from continuity arguments, as both the origin (8) and the result (10) of (9) are obviously continuous in $a_{i},$ and a set with equivalent nuclei can be obtained as a limit of a set with distinct nuclei.

Equations (8)–(10) have one zero root mentioned above, corresponding to eigenvector of all ones obtained by lowering the projection of the function with the maximum possible total spin and projection

(11) $$|J=\frac{n+1}{2},K=\frac{n+1}{2}\rangle =|+;+++\cdots +\rangle ,\;|J,K-1\rangle \propto J_{-}|J,K\rangle \propto \sum_{i=0}^{n}|\xi_{i}\rangle ,$$

all the remaining n roots are non-zero and positive. If all coupling constants are distinct, $0<a_{1}<a_{2}<\cdots <a_{n},$ all $\lambda_{k}$ are also distinct and are separated by consecutive $a_{i}$ as

(12) $$a_{1}<\lambda_{1}<a_{2}<\lambda_{2}<a_{3}<\lambda_{3}<\cdots <a_{n-1}<\lambda_{n-1}<a_{n}<\lambda_{n},$$

and all the corresponding eigenvectors are fully entangled, i.e., all their coordinates in the basis (3) are non-zero. This case corresponds to the last line in (9). If equivalent nuclei with multiple equal coupling constants are allowed, and a radical has n spin-12 nuclei with constants $0<a_{1}<a_{2}<\cdots <a_{r},\;r\leq n$ with multiplicities $m_{i}\geq 1,\;i=1,\;2,\;\ldots ,\;r\leq n$, the n+1 roots of the characteristic polynomial are organized as follows:

(13) $$\begin{array}{l} \lambda_{0}=0;\\ \mathrm{for}\;\mathrm{each}\;m_{i}>1:\;{\lambda^{\prime }}_{i}=a_{i}\;\mathrm{of}\;\mathrm{multiplicity}\;m_{i}-1;\\ \mathrm{the}\;\mathrm{remaining}\;r\;\mathrm{roots}\;\lambda_{i}\;\mathrm{satisfy}:\;\\ \;\;a_{1}<\lambda_{1}<a_{2}<\lambda_{2}<\cdots <a_{r-1}<\lambda_{r-1}<a_{r}<\lambda_{r}. \end{array}$$

The roots ${\lambda^{\prime }}_{i}=a_{i}$ appearing for each multiple constant give rise to the ambiguity in (9) mentioned above and related to formal division by zero compensated by multiplication with zero pre-factor, but analysis showed that all three forms are valid for an arbitrary set of hyperfine coupling constants.

In practice the most useful of the three is the polynomial form (10), where when writing $A_{i}$ the constants $a_{i}$ should be counted for each of n nuclei considering the multiplicities. Still this is an *n*-th order algebraic equation with up to n free parameters $A_{i}$ equivalent to n original free coupling constants $a_{i}.$ The apparent simplicity of the polynomial in (10) is deceptive, and although only regular numeric prefactors (k+1) in each term $(k+1)A_{k}{\left(-\lambda \right)}^{n-k}$ distinguish it from the trivial equation having $\lambda_{i}=a_{i}$ by Vieta’s formulae, these “simple numbers” lead to catastrophic consequences in terms of complexity. The following sections will show how this problem can be at least partially overcome by geometric visualization.

### 2.2. The Zero-Splitting Transform

The matrix (5), although written in a very symmetric form, has a zero eigenvalue with the known eigenvector of all ones. The zero $\lambda_{0}$ is an inconvenience, as it makes the matrix degenerate and limits possible manipulations with it, while the known eigenvector suggests that the subspace that it spans can be split away. In this section we shall construct such a zero-splitting transform.

We need an unitary transform to another (n+1)-dimensional basis, of which one vector is already known:

(14) $$|\phi_{0}\rangle ={\left(1,\;1,\;\ldots ,\;1,1\right)}^{T}/\sqrt{n+1},$$

and we want the remaining n vectors to reflect the equal roles of all n nuclei, so that the new basis retains this symmetry. A convenient and regular way of building such a basis is to generate it by consecutive powers of n distinct *n*-th roots of unity $\varepsilon_{k}=\varepsilon^{k},$ where ε=exp(2πi/n) is the *n*-th root of unity with the smallest positive argument, as

(15) $$|\phi_{k+1}\rangle =u_{k}{\left(w_{k},\;1,\;\varepsilon_{k},\;\varepsilon_{k}^{2},\;\ldots ,\;\varepsilon_{k}^{n-1}\right)}^{T},\;k=0,\;\ldots ,\;n-1,$$

where the first element and the normalizing prefactor in each vector have to be chosen to ensure orthonormality of the entire set. The corresponding unitary transform matrix $C_{1}$ has the form

(16) $$C_{1}=\frac{1}{\sqrt{n(n+1)}}\left(\begin{array}{cccccc} \sqrt{n} & -n & 0 & 0 & \cdots & 0\\ \sqrt{n} & 1 & \sqrt{n+1} & \sqrt{n+1} & \cdots & \sqrt{n+1}\\ \sqrt{n} & 1 & \varepsilon \sqrt{n+1} & \varepsilon^{2}\sqrt{n+1} & \cdots & \varepsilon^{n-1}\sqrt{n+1}\\ \sqrt{n} & 1 & \varepsilon^{2}\sqrt{n+1} & {\left(\varepsilon^{2}\right)}^{2}\sqrt{n+1} & \cdots & {\left(\varepsilon^{n-1}\right)}^{2}\sqrt{n+1}\\ \vdots & \vdots & \vdots & \vdots & \ddots & \vdots \\ \sqrt{n} & 1 & \varepsilon^{n-1}\sqrt{n+1} & {\left(\varepsilon^{2}\right)}^{n-1}\sqrt{n+1} & \cdots & {\left(\varepsilon^{n-1}\right)}^{n-1}\sqrt{n+1} \end{array}\right),$$

and taking the transform by multiplying out $H_{1}=C_{1}^{+}HC_{1},$ we obtain

(17) $$H_{1}=\frac{1}{n}\left(\begin{array}{cccccc} 0 & 0 & 0 & 0 & \cdots & 0\\ 0 & (n+1)A\left(1\right) & \sqrt{n+1}A\left(\varepsilon \right) & \sqrt{n+1}A\left(\varepsilon^{2}\right) & \cdots & \sqrt{n+1}A\left(\varepsilon^{n-1}\right)\\ 0 & \sqrt{n+1}A\left(\varepsilon^{n-1}\right) & A\left(1\right) & A\left(\varepsilon \right) & \cdots & A\left(\varepsilon^{n-2}\right)\\ 0 & \sqrt{n+1}A\left(\varepsilon^{n-2}\right) & A\left(\varepsilon^{n-1}\right) & A\left(1\right) & \cdots & A\left(\varepsilon^{n-3}\right)\\ \vdots & \vdots & \vdots & \vdots & \ddots & \vdots \\ 0 & \sqrt{n+1}A\left(\varepsilon \right) & A\left(\varepsilon^{2}\right) & A\left(\varepsilon^{3}\right) & \cdots & A\left(1\right) \end{array}\right),$$

where the following complex combinations of parameters $a_{i}$ weighted with consecutive powers of the corresponding root of unity have been introduced:

(18) $$A\left(\varepsilon^{k}\right)=a_{1}+\varepsilon^{k}a_{2}+{\left(\varepsilon^{k}\right)}^{2}a_{3}+\ldots +{\left(\varepsilon^{k}\right)}^{n-1}a_{n},\;k=0,\;\ldots ,\;n-1.$$

We have indeed split off the subspace spanned by the eigenvector of $\lambda_{0}=0$ by obtaining the zero “hook” in the transformed matrix and a smaller n×n but explicitly complex Hermitian matrix that is in its way symmetric with respect to all n nuclei. It can also be seen that the second function of the new basis $|\phi_{2}\rangle ={\left(-n,\;1,\;1,\;\ldots ,\;1,\;1\right)}^{T}/\sqrt{n\left(n+1\right)}$ stands out from the set (15), as coefficients for elements in the second row and second column are larger than for other nonzero rows and columns. This is a reflection of the entanglement of the eigenfunctions of Hamiltonian (5) mentioned in the previous Section: at least for distinct nuclei they all must contain a nonzero contribution from function $|\xi_{0}\rangle =|-;+++\cdots +\rangle$ of the set (3), that is, their first component must be non-zero. However, the only basis vector of the new set (15) with non-zero first component is $|\phi_{2}\rangle ,$ which endows it with a special role.

In splitting away the zero eigenvalue we have obtained a smaller complex matrix (17), but sacrificed the real symmetry of the original matrix (5). This can be restored for the smaller matrix by making another unitary transform and “shuffling back” the basis in the following way. Again, use complex vectors (15) to generate a new basis, but this time confine it only to the subspace with nonzero eigenvalues leaving alone the split-off one-dimensional subspace. Arrange it in the columns of an unitary matrix as

(19) $$C_{2}=\frac{1}{\sqrt{n}}\left(\begin{array}{cccccc} \sqrt{n} & 0 & 0 & 0 & \cdots & 0\\ 0 & 1 & 1 & 1 & \cdots & 1\\ 0 & 1 & \varepsilon & \varepsilon^{2} & \cdots & \varepsilon^{n-1}\\ 0 & 1 & \varepsilon^{2} & {\left(\varepsilon^{2}\right)}^{2} & \cdots & {\left(\varepsilon^{n-1}\right)}^{2}\\ \vdots & \vdots & \vdots & \vdots & \ddots & \vdots \\ 0 & 1 & \varepsilon^{n-1} & {\left(\varepsilon^{2}\right)}^{n-1} & \cdots & {\left(\varepsilon^{n-1}\right)}^{n-1} \end{array}\right),$$

and apply the transform backwards by multiplying out $H_{2}=C_{2}^{}H_{1}C_{2}^{+}$ to obtain

(20) $$H_{2}=\frac{1}{n^{2}}\left(\begin{array}{cccccc} 0 & 0 & 0 & 0 & \cdots & 0\\ 0 & A_{1}\gamma +a_{1}\delta & A_{1}\gamma +\left(a_{1}+a_{2}\right)\chi & A_{1}\gamma +\left(a_{1}+a_{3}\right)\chi & \cdots & A_{1}\gamma +\left(a_{1}+a_{n}\right)\chi \\ 0 & A_{1}\gamma +\left(a_{2}+a_{1}\right)\chi & A_{1}\gamma +a_{2}\delta & A_{1}\gamma +\left(a_{2}+a_{3}\right)\chi & \cdots & A_{1}\gamma +\left(a_{2}+a_{n}\right)\chi \\ 0 & A_{1}\gamma +\left(a_{3}+a_{1}\right)\chi & A_{1}\gamma +\left(a_{3}+a_{2}\right)\chi & A_{1}\gamma +a_{3}\delta & \cdots & A_{1}\gamma +\left(a_{3}+a_{n}\right)\chi \\ \vdots & \vdots & \vdots & \vdots & \ddots & \vdots \\ 0 & A_{1}\gamma +\left(a_{n}+a_{1}\right)\chi & A_{1}\gamma +\left(a_{n}+a_{2}\right)\chi & A_{1}\gamma +\left(a_{n}+a_{3}\right)\chi & \cdots & A_{1}\gamma +a_{n}\delta \end{array}\right)$$

with

(21) $$\gamma ={\left(\sqrt{n+1}-1\right)}^{2};\;\delta =n\left(2\sqrt{n+1}+n-2\right);\;\chi =n\left(\sqrt{n+1}-1\right).$$

The real orthogonal transform that produces $H_{2}$ directly from H given by the matrix product $C_{1}C_{2}^{+}$ has the form

(22) $$C_{12}=\frac{1}{n\sqrt{n+1}}\left(\begin{array}{cccccc} n & -n & -n & -n & \cdots & -n\\ n & \alpha & \beta & \beta & \cdots & \beta \\ n & \beta & \alpha & \beta & \cdots & \beta \\ n & \beta & \beta & \alpha & \cdots & \beta \\ \vdots & \vdots & \vdots & \vdots & \ddots & \vdots \\ n & \beta & \beta & \beta & \cdots & \alpha \end{array}\right)$$

with

(23) $$\alpha =1+(n-1)\sqrt{n+1};\;\beta =1-\sqrt{n+1}.$$

If only the real split-zero transformed matrix is needed, the matrix (20) can be obtained directly from (5) using real-only matrices by taking the transform $H_{2}=C_{12}^{+}HC_{12}^{}$ and completely bypassing three complex matrices in (16)–(19). We also note that the suggested transforms have turned a sparse matrix (5) into dense but regular matrices using also dense transform matrices: while the starting matrix had non-zero elements only at diagonal and in the hook, the transformed matrices have not identically zero elements everywhere but in the hook.

The transforms (16) and (20) are unitary by construction and preserve the spectrum of the transformed matrix, therefore the matrices (17) and (20) have equation (10) as their characteristic polynomial. The zero root corresponds to the zero hook of the first row and first column, and all the positive roots are inherited by the smaller positive definite n×n matrices that have the characteristic polynomial

(24) $$f_{n}(\lambda )={\left(-\lambda \right)}^{n}+2A_{1}{\left(-\lambda \right)}^{n-1}+3A_{2}{\left(-\lambda \right)}^{n-2}+4A_{3}{\left(-\lambda \right)}^{n-3}+\cdots +\left(n+1\right)A_{n}.$$

The traces of matrices (17) and (20) are equal to $2A_{1},$ the coefficient at the second highest degree of the characteristic polynomial, and determinants of the n×n matrices are equal to $\left(n+1\right)A_{n},$ the free term. In the following sections we shall omit the zero hook and work with the obtained n×n matrices, referring to them as the result of the zero-splitting transform of this section. The natural ordering of the basis vectors generated by *n*-th roots of unity given above will be used throughout.

### 2.3. The Real Symmetric Zero-Split Matrix 

The n×n real symmetric matrix in (20) is useful for making a connection to the well-known reference case of all equivalent nuclei and introducing circulants. Omitting the zero hook and setting all $a_{i}=a,$ we obtain the following n×n matrix:

(25) $$N=a\left(\begin{array}{ccccc} 2 & 1 & 1 & \cdots & 1\\ 1 & 2 & 1 & \cdots & 1\\ 1 & 1 & 2 & \cdots & 1\\ \vdots & \vdots & \vdots & \ddots & \vdots \\ 1 & 1 & 1 & \cdots & 2 \end{array}\right).$$

It can be seen that all its rows are generated by a single line (2, 1, 1, …, 1) circularly shifted one position to the right when going one row down. The eigenvalues of such a matrix, which will be our roots for the case of *n* equivalent nuclei, are found by guessing its eigenvectors based on this circulant symmetry: these are again given by consecutive powers of an *n*-th root of unity. It can be seen that the system of linear equations

(26) $$a\left(\begin{array}{ccccc} 2 & 1 & 1 & \cdots & 1\\ 1 & 2 & 1 & \cdots & 1\\ 1 & 1 & 2 & \cdots & 1\\ \vdots & \vdots & \vdots & \ddots & \vdots \\ 1 & 1 & 1 & \cdots & 2 \end{array}\right)\left(\begin{array}{c} \varepsilon \\ \varepsilon^{2}\\ \varepsilon^{3}\\ \vdots \\ \varepsilon^{n}=1 \end{array}\right)=\lambda \left(\begin{array}{c} \varepsilon \\ \varepsilon^{2}\\ \varepsilon^{3}\\ \vdots \\ \varepsilon^{n}=1 \end{array}\right)$$

is satisfied identically, as multiplying the first equation by ε produces the second equation and so on, and the last equation multiplied by ε gives again the first one, closing the circulant pattern across the rows. Taking n different *n*-th roots of unity $\varepsilon_{k},\;k=0,\;\ldots ,\;n-1,$ we thus obtain n different orthogonal eigenvectors, and the corresponding eigenvalue is directly read off from the last equation and is given by

(27) $$\lambda_{k}=a\left(\varepsilon_{k}+\varepsilon_{k}^{2}+\ldots +\varepsilon_{k}^{n-1}+2\right)=a\left(\varepsilon^{k}+\varepsilon^{2k}+\ldots +\varepsilon^{\left(n-1\right)k}+2\right),\;k=0,\ldots ,n-1.$$

Since for *n*-th roots of unity $\varepsilon_{k}$

(28) $$\sum_{m=0}^{n-1}\varepsilon_{k}^{m}=\frac{1-\varepsilon_{k}^{n}}{1-\varepsilon_{k}}=\{\begin{array}{c} n,\;\varepsilon_{k}=1\;(k=0)\\ 0,\;\varepsilon_{k}\neq 1\;(k=1,\;\ldots ,\;n-1) \end{array},$$

the eigenvalues of Equation (26) are

(29) $$\begin{array}{l} \lambda =a,\;\mathrm{multiplicity}\;n-1,\\ \lambda =(n+1)a,\;\mathrm{multiplicity}\;1. \end{array}$$

This dichotomy of obtaining either 1 or n+1 depending on whether summing all consecutive powers of ε=1 or ε≠1 will be met in numerous places. The special role of the basis vector corresponding to ε=1 (and thus comprised of all ones) as opposed to vectors generated by other roots of unity ε≠1 is also encountered quite often.

The obtained roots are consistent with the results (13), giving λ=a with multiplicity n−1 and a nondegenerate root λ>a for n equivalent nuclei. The obtained roots are also consistent with energy levels that can be directly obtained from consideration of spin Hamiltonian (1) in case of n equivalent spin-12 nuclei with restriction to subspace with total spin projection $K_{\max}-1.$ The Hamiltonian and its energies in this case read

(30) H^=aS→·I→Σ, Ek=aJ(J+1)−IΣ(IΣ+1)−342, J=IΣ±12.

Three types of states can enter the considered subspace with $K=K_{\max}-1.$ One of these is the state with $I_{\Sigma }=I_{\max}=n/2$ and J=IΣ+12=(n+1)/2, i.e., the function (11) from the multiplet with maximum possible total spin that gave us the zero root split away in the preceding section. The others are one state with $I_{\Sigma }=I_{\max}=n/2$ and J=IΣ−12=(n−1)/2, and n−1 states with $I_{\Sigma }=I_{\max}-1=n/2-1$ and J=IΣ+12=(n−1)/2, the latter corresponding to n−1 possible ways of combining n nuclear spins-12 into total nuclear spin $I_{\Sigma }=I_{\max}-1=n/2-1$ with maximum projection, i.e., to n−1 possible nuclear multiplets with $I_{\Sigma }=n/2-1.$ Taking into account the connection (6) between the roots $\lambda_{k}$ and energies $E_{k},$ we obtain Table 1 that reproduces (29).

The characteristic polynomial (24) factors into a much simpler form

(31) $$f_{n}\left(\lambda \right)={\left(\lambda -a\right)}^{n-1}\left(\lambda -(n+1)a\right).$$

On the other hand, knowing from the general properties that for n equivalent nuclei we must have n−1 roots equal to a leaves only one unknown λ to figure out, and this can be done by considering the simplest possible relation connecting the sum of all roots with the coefficient at the second highest degree of λ

(32) $$\sum_{i}\lambda_{i}=(n-1)a+\lambda =2A_{1}=2na\Rightarrow \lambda =(n+1)a.$$

In fact, it suffices to know that we must have a degenerate root $\lambda_{1}$ with multiplicity n−1 and a non-degenerate root $\lambda_{2},$ and consider the relations for the trace and determinant to obtain the sought roots: it can be seen that the system of equations

(33) $$\begin{array}{l} \sum_{i}\lambda_{i}=(n-1)\lambda_{1}+\lambda_{2}=2A_{1}=2na\\ \prod_{i}\lambda_{i}=\lambda_{1}^{n-1}\lambda_{2}=(n+1)A_{n}=(n+1)a^{n} \end{array}$$

is satisfied with $\lambda_{1}=a$ and $\lambda_{2}=\left(n+1\right)a$.

Not much can be done with the real symmetric matrix to move beyond the equivalent nuclei, as the shifting pattern symmetry of the type seen in (20) is lost when the couplings become not all equal, and the circulant property is thus lost as well. However, further advances are possible with the complexified matrix of (17) that upon inspection also shows a form of circulant symmetry, but without reference to specific coupling constants $a_{i},$ which are now hidden inside the complex “coupling constants” $A\left(\varepsilon^{k}\right)$.

### 2.4. The Complexified Coupling Constants

The complexified “coupling constants” $A\left(\varepsilon^{k}\right)$ defined by Equation (18) are the third form of parametrizing the problem of finding the roots with the input parameters of the original physical problem, the hyperfine coupling constants $a_{i}.$ The set $\left\{a_{i}\right\}$ itself is the first and obvious parametrization used in the Hamiltonian (1) and its matrix representation (5). The second one is the set $\left\{A_{i}\right\}$ defined by (7), which is natural for the polynomial representation, but is not linearly related to $\left\{a_{i}\right\}.$ The set $\left\{A(\varepsilon^{k})\right\}$ can be viewed as a result of transforming the “vector” consisting of $a_{i}$ with an unitary matrix built from vectors again generated by consecutive powers of *n*-th roots of unity:

(34) $$\left(\begin{array}{c} A\left(1\right)\\ A\left(\varepsilon \right)\\ A\left(\varepsilon^{2}\right)\\ \vdots \\ A\left(\varepsilon^{n-1}\right) \end{array}\right)=\left(\begin{array}{ccccc} 1 & 1 & 1 & \cdots & 1\\ 1 & \varepsilon & \varepsilon^{2} & \cdots & \varepsilon^{n-1}\\ 1 & \varepsilon^{2} & {\left(\varepsilon^{2}\right)}^{2} & \cdots & {\left(\varepsilon^{2}\right)}^{n-1}\\ 1 & \vdots & \vdots & \ddots & \vdots \\ 1 & \varepsilon^{n-1} & {\left(\varepsilon^{n-1}\right)}^{2} & \cdots & {\left(\varepsilon^{n-1}\right)}^{n-1} \end{array}\right)\left(\begin{array}{c} a_{1}\\ a_{2}\\ a_{3}\\ \vdots \\ a_{n} \end{array}\right)=\sqrt{n}C_{3}^{+}\left(\begin{array}{c} a_{1}\\ a_{2}\\ a_{3}\\ \vdots \\ a_{n} \end{array}\right),$$

where $C_{3}$ is the unitary matrix and the $\sqrt{n}$ multiplier has been added since the vectors corresponding to the original definition of (18) were not normalized. As opposed to the set $\left\{A_{i}\right\},$ the mapping $\left\{a_{i}\right\}\to \left\{A\left(\varepsilon^{k}\right)\right\}$ is linear bijective and can be easily reversed if needed using the Hermitian conjugate of matrix from (34) and dividing the result by n. The inverse relation has the form

(35) $$\left(\begin{array}{c} a_{1}\\ a_{2}\\ a_{3}\\ \vdots \\ a_{n} \end{array}\right)=\frac{1}{n}\left(\begin{array}{ccccc} 1 & 1 & 1 & \cdots & 1\\ 1 & \bar{\varepsilon } & {\bar{\varepsilon }}^{2} & \cdots & {\bar{\varepsilon }}^{n-1}\\ 1 & {\bar{\varepsilon }}^{2} & {\left({\bar{\varepsilon }}^{2}\right)}^{2} & \cdots & {\left({\bar{\varepsilon }}^{2}\right)}^{n-1}\\ 1 & \vdots & \vdots & \ddots & \vdots \\ 1 & {\bar{\varepsilon }}^{n-1} & {\left({\bar{\varepsilon }}^{n-1}\right)}^{2} & \cdots & {\left({\bar{\varepsilon }}^{n-1}\right)}^{n-1} \end{array}\right)\left(\begin{array}{c} A\left(1\right)\\ A\left(\varepsilon \right)\\ A\left(\varepsilon^{2}\right)\\ \vdots \\ A\left(\varepsilon^{n-1}\right) \end{array}\right)=\frac{C_{3}}{\sqrt{n}}\left(\begin{array}{c} A\left(1\right)\\ A\left(\varepsilon \right)\\ A\left(\varepsilon^{2}\right)\\ \vdots \\ A\left(\varepsilon^{n-1}\right) \end{array}\right).$$

A useful view of the mapping $\left\{a_{i}\right\}\to \left\{A\left(\varepsilon^{k}\right)\right\}$ is interpreting the original set $\left\{a_{i}\right\}$ as a function of an evenly spaced discrete argument {i}. Then the transform (34) is recognized as a discrete Fourier transform (DFT) for the “function” $\left\{a_{i}\right\},$ and transform (35) is inverse DFT, which opens a connection to the vast mathematical field of fast Fourier transforms [104] and more algebraically abstract subject of Fourier transform in finite groups [105] and Gaussian sums over finite fields [106]. FFT is widely used in signal processing for efficient manipulation of polynomials with coefficients $\left\{a_{i}\right\}$ [107], and is not directly employed here, still it is a huge resource to draw from.

A useful bit of intuition from the connection with Fourier transform is that the slower the variation of the function, the faster its Fourier coefficients decay. Thus, a useful property of the set $\left\{A(\varepsilon^{k})\right\}$ is that for equivalent nuclei all $A\left(\varepsilon^{k}\right)$ vanish identically except for $A\left(1\right)=na=A_{1}$ as a direct consequence of the sum rule (28), since for equivalent nuclei $A\left(\varepsilon^{k}\right)$ turns simply into the sum of all powers of $\varepsilon^{k}$ times the coupling constant. The matrix (17) (with omitted zero hook) then turns into a diagonal one

(36) $$H_{eq}=a\times \mathrm{diag}\left\{n+1,\;1,\;1,\;\ldots ,\;1,\;1\right\},$$

reproducing eigenvalues (29) and factored characteristic polynomial (31). For small deviations from equivalence the matrix will be almost diagonal, with first superdiagonal and subdiagonal given by small A(ε) and its complex conjugate $A\left(\varepsilon^{n-1}\right),$ respectively, and progressively farther super-/subdiagonals given by further consecutive components of the Fourier transform $\left\{A(\varepsilon^{k})\right\}$ that will progressively decay if variations in the original set $\left\{a_{i}\right\}$ are modest. Thus, the complex split-zero matrix is natural for describing the loss of equivalence. However, before doing this, one additional step will be useful.

### 2.5. The Scaling Transform

Let us take the complex split-zero matrix (17), omit the zero hook, and denote the resulting n×n matrix as M:

(37) $$M=\frac{1}{n}\left(\begin{array}{ccccc} (n+1)A\left(1\right) & \sqrt{n+1}A\left(\varepsilon \right) & \sqrt{n+1}A\left(\varepsilon^{2}\right) & \cdots & \sqrt{n+1}A\left(\varepsilon^{n-1}\right)\\ \sqrt{n+1}A\left(\varepsilon^{n-1}\right) & A\left(1\right) & A\left(\varepsilon \right) & \cdots & A\left(\varepsilon^{n-2}\right)\\ \sqrt{n+1}A\left(\varepsilon^{n-2}\right) & A\left(\varepsilon^{n-1}\right) & A\left(1\right) & \cdots & A\left(\varepsilon^{n-3}\right)\\ \vdots & \vdots & \vdots & \ddots & \vdots \\ \sqrt{n+1}A\left(\varepsilon \right) & A\left(\varepsilon^{2}\right) & A\left(\varepsilon^{3}\right) & \cdots & A\left(1\right) \end{array}\right).$$

As mentioned earlier, M has inherited all the nontrivial positive roots for the original problem and has (24) as its characteristic polynomial. The form of matrix in (37) invites to re-scale the first basis vector as $|\phi_{1}\rangle \to |\phi_{1}\rangle /\sqrt{n+1},$ which will remove the factors (n+1) and $\sqrt{n+1}$ in the corresponding matrix elements of the first line and first column. Such a transformation is equivalent to matrix transform

(38) $$M\to M_{1}=C_{4}^{-1}MC_{4}^{-1},\;C_{4}^{-1}=\mathrm{diag}\left\{\frac{1}{\sqrt{n+1}},1,\;1,\;\ldots ,\;1\right\}$$

to produce

(39) $$M_{1}=\frac{1}{n}\left(\begin{array}{ccccc} A\left(1\right) & A\left(\varepsilon \right) & A\left(\varepsilon^{2}\right) & \cdots & A\left(\varepsilon^{n-1}\right)\\ A\left(\varepsilon^{n-1}\right) & A\left(1\right) & A\left(\varepsilon \right) & \cdots & A\left(\varepsilon^{n-2}\right)\\ A\left(\varepsilon^{n-2}\right) & A\left(\varepsilon^{n-1}\right) & A\left(1\right) & \cdots & A\left(\varepsilon^{n-3}\right)\\ \vdots & \vdots & \vdots & \ddots & \vdots \\ A\left(\varepsilon \right) & A\left(\varepsilon^{2}\right) & A\left(\varepsilon^{3}\right) & \cdots & A\left(1\right) \end{array}\right).$$

This scaling transform is not unitary, and it does not preserve the spectrum. However, it changes the problem in a manageable and, in this place, in a convenient way. We also note that the scaling transform has modified the trace and determinant of the complex zero-split matrix M in the following way:

(40) $$\mathrm{Tr}\left(M_{1}\right)=\frac{1}{2}\mathrm{Tr}\left(M\right)=A_{1},\;\left|M_{1}\right|=\frac{1}{n+1}\left|M\right|=\prod_{i}a_{i}.$$

The first relation is seen directly from the definition of $M_{1}$ in (39), and the second one comes from taking determinants at both sides of the scaling transform (38) and taking $\left|M\right|=\left(n+1\right)A_{n}$ as the last term in characteristic polynomial (24).

### 2.6. The Loss of Equivalence as a Perturbation

Let us take the system of equations for the eigensystem of matrix (37) $M\vec{c}=\lambda \vec{c}$ and scale it using the transform (38) by writing the following sequence of determinant identities:

(41) $$\left|M-\lambda \hat{1}\right|=0=\left|C_{4}^{-1}\right|\left|M-\lambda \hat{1}\right|\left|C_{4}^{-1}\right|=\left|C_{4}^{-1}\left(M-\lambda \hat{1}\right)C_{4}^{-1}\right|=\left|M_{1}-\lambda C_{4}^{-2}\right|=\left|M_{1}-\lambda {\hat{1}}_{1}\right|,$$

where

(42) $${\hat{1}}_{1}=\mathrm{diag}\left\{\frac{1}{n+1},1,1,\ldots ,1\right\}$$

is the modified identity matrix with first element scaled down, to obtain the equation $M_{1}\vec{c}=\lambda {\hat{1}}_{1}\vec{c}:$

(43) M1c→=1n(A(1)A(ε)A(ε2)⋯A(εn−1)A(εn−1)A(1)A(ε)⋯A(εn−2)A(εn−2)A(εn−1)A(1)⋯A(εn−3)⋮⋮⋮⋱⋮A(ε)A(ε2)A(ε3)⋯A(1))(c1c2c3⋮cn)=λ(c1n+1c2c3⋮cn).

Taking all nuclei to be equivalent, we have $A\left(1\right)=na,\;A\left(\varepsilon^{k}\neq 1\right)=0,$ and (43) becomes

(44) a(100⋯0010⋯0001⋯0⋮⋮⋮⋱⋮000⋯1)(c1c2c3⋮cn)=λ(c1n+1c2c3⋮cn).

This equation has unit vectors ${\vec{e}}_{k}$ having only one 1 in the *k*-th position, k=1,…,n, as its solution (“eigenvectors”), and has one non-degenerate root λ=(n+1)a with eigenvector ${\vec{e}}_{1}$ and (n−1)-degenerate root λ=a with eigenvectors ${\vec{e}}_{k},$ k=2,…,n. This is of course again the solution for equivalent nuclei (29), but in a much more convenient way to apply the equivalence-lifting perturbation, because algebraically the problem now is to apply a perturbation to identity-like matrix, a well-developed topic covered in matrix theory books [108]. We shall only discuss first-order perturbation here, underlining its similarity to conventional quantum mechanical perturbation theory. 

Let us introduce a deviation from equivalence by shifting the coupling constants as $a_{i}=a+\delta a_{i}.$ This induces the corresponding shifts to complexified coupling constants

(45) $$\delta A\left(\varepsilon^{k}\right)=\delta a_{1}+\varepsilon^{k}\delta a_{2}+{\left(\varepsilon^{k}\right)}^{2}\delta a_{3}+\ldots +{\left(\varepsilon^{k}\right)}^{n-1}\delta a_{n},\;k=0,\;\ldots ,\;n-1.$$

Equation (43) up to first order in perturbation can be written as

(46) $$\left(M_{1}^{0}+\delta M_{1}\right)\left({\vec{c}}_{i}^{0}+\delta {\vec{c}}_{i}\right)=\left(\lambda_{i}^{0}+\delta \lambda_{i}\right){\hat{1}}_{1}\left({\vec{c}}_{i}^{0}+\delta {\vec{c}}_{i}\right),$$

where $M_{1}^{0}$ is the matrix from (44), $\delta M_{1}$ is the matrix from (43) with all $A\left(\varepsilon^{k}\right),\;k=0,\ldots ,n-1$ changed to the corresponding $\delta A\left(\varepsilon^{k}\right),$ and unperturbed eigenvectors and eigenvalues are the solution of (44). As in quantum mechanics, we have two subproblems, for single non-degenerate state with ${\vec{c}}_{1}^{0}={\vec{e}}_{1},\;\lambda_{1}^{0}=(n+1)a,$ and for a group of n−1 degenerate states with ${\vec{c}}_{k}^{0}={\vec{e}}_{k},\;k=2,\;\ldots ,\;n,\;\lambda_{k}^{0}=a$.

Treating first the non-degenerate case and omitting the subscript for simplicity, we must have

(47) $$\delta \vec{c}={\left(0,\;\delta c_{2},\;\delta c_{3},\;\ldots ,\;\delta c_{n}\right)}^{T},$$

with first component equal to zero to keep the perturbed vector ${\vec{c}}_{}^{0}+\delta \vec{c}$ normalized to 1 to first order. Equation (46) is satisfied identically in zero order, and in the first order we obtain

(48) $$M_{1}^{0}\delta \vec{c}+\delta M_{1}{\vec{c}}_{}^{0}=\lambda_{}^{0}E_{1}\delta \vec{c}+\delta \lambda {\hat{1}}_{1}{\vec{c}}_{}^{0}.$$

Expanding, this gives

(49) $$a\left(\begin{array}{c} 0\\ \delta c_{1}\\ \delta c_{2}\\ \vdots \\ \delta c_{n} \end{array}\right)+\frac{1}{n}\left(\begin{array}{c} \delta A\left(1\right)\\ \delta A\left(\varepsilon^{n-1}\right)\\ \delta A\left(\varepsilon^{n-2}\right)\\ \vdots \\ \delta A\left(\varepsilon \right) \end{array}\right)=(n+1)a\left(\begin{array}{c} 0\\ \delta c_{2}\\ \delta c_{3}\\ \vdots \\ \delta c_{n} \end{array}\right)+\delta \lambda \left(\begin{array}{c} \frac{1}{n+1}\\ 0\\ 0\\ \vdots \\ 0 \end{array}\right).$$

As expected, the terms with δλ and $\delta c_{i}$ separate, and we obtain the first-order corrections to the non-degenerate root and its eigenvector:

(50) $$\delta \lambda =\frac{n+1}{n}\delta A\left(1\right),\;\left(\begin{array}{c} \delta c_{1}\\ \delta c_{2}\\ \delta c_{4}\\ \vdots \\ \delta c_{n} \end{array}\right)=\frac{1}{n^{2}a}\left(\begin{array}{c} 0\\ \delta A\left(\varepsilon^{n-1}\right)\\ \delta A\left(\varepsilon^{n-2}\right)\\ \vdots \\ \delta A\left(\varepsilon \right) \end{array}\right)=\frac{1}{n^{2}a}\left(\begin{array}{c} 0\\ \delta A\left(\bar{\varepsilon }\right)\\ \delta A\left({\bar{\varepsilon }}^{2}\right)\\ \vdots \\ \delta A\left({\bar{\varepsilon }}^{n-1}\right) \end{array}\right).$$

It can be seen that the correction to the root, i.e., to the energy of the eigenstate, depends only on $\delta A\left(1\right)=\delta A_{1}=\sum_{i}\delta a_{i},$ i.e., on the shift of the center of gravity of the set of coupling constants, while the correction to the function depends on $\delta A\left(\varepsilon^{k}\right),$ i.e., on lifting the degeneracy of the set of coupling constants. 

Since the state remains non-degenerate, its function must be explicitly real, and this is indeed so. The correction $\delta \vec{c}$ to the state vector written in basis $\left\{|\eta_{i}\rangle \right\}$ means that the function is corrected by $|\delta \psi \rangle =\sum_{i}\delta c_{i}|\eta_{i}\rangle .$ We recall that the complex split-zero matrix M is written in complexified shuffled basis of type (15), of which the needed part $\left\{|\eta_{k}\rangle \right\},k=2,\;\ldots ,\;n$ can be expressed via original basis (3) with an (n−1)×n matrix as

(51) $$\left(\begin{array}{c} |\eta_{2}\rangle \\ |\eta_{3}\rangle \\ |\eta_{4}\rangle \\ \vdots \\ |\eta_{n}\rangle \end{array}\right)=\frac{1}{\sqrt{n}}\left(\begin{array}{ccccc} 1 & \varepsilon & \varepsilon^{2} & \cdots & \varepsilon^{n-1}\\ 1 & \varepsilon^{2} & {\left(\varepsilon^{2}\right)}^{2} & \cdots & {\left(\varepsilon^{2}\right)}^{n-1}\\ 1 & \varepsilon^{3} & {\left(\varepsilon^{3}\right)}^{2} & \cdots & {\left(\varepsilon^{3}\right)}^{n-1}\\ 1 & \vdots & \vdots & \ddots & \vdots \\ 1 & \varepsilon^{n-1} & {\left(\varepsilon^{n-1}\right)}^{2} & \cdots & {\left(\varepsilon^{n-1}\right)}^{n-1} \end{array}\right)\left(\begin{array}{c} |\xi_{1}\rangle \\ |\xi_{2}\rangle \\ |\xi_{3}\rangle \\ \vdots \\ |\xi_{n}\rangle \end{array}\right).$$

Taking $\delta c_{i}$ from (50) and $|\eta_{i}\rangle$ from (51), after rearrangement we obtain the following result for the correction and the original unperturbed function:

(52) $$\begin{array}{l} |\delta \psi_{1}\rangle =\sum_{i=2}^{n}\delta c_{i}|\eta_{i}\rangle =\frac{n-1}{n^{2}\sqrt{n}a}\sum_{k=1}^{n}|\xi_{k}\rangle \delta a_{k}-\frac{1}{n^{2}\sqrt{n}a}\sum_{\begin{array}{l} k,l=1\\ \;k\neq l \end{array}}^{n}|\xi_{k}\rangle \delta a_{l},\\ |\psi_{1}^{0}\rangle =\frac{1}{\sqrt{n+1}}\left(-n|\xi_{0}\rangle +\sum_{k=1}^{n}|\xi_{k}\rangle \right). \end{array}$$

Note that the function of the original basis with flipped electron spin $|\xi_{0}\rangle =|-;++\cdots +\rangle$ is present only in the unperturbed function $|\psi_{1}^{0}\rangle$ and does not enter the perturbative correction $|\delta \psi_{1}\rangle$ that only redistributes the functions $\left\{|\xi_{k}\rangle \right\},k=2,\;\ldots ,\;n$.

Turning to the problem of the (n−1)-degenerate subspace, we first note that this subspace was not modified by the scaling transform (38), and therefore the problem reduces to the ordinary quantum mechanical degenerate perturbation theory, i.e., to finding the eigenvalues and eigenvectors of the perturbation matrix restricted to the degenerate subspace. Since the basis of this subspace is just the set of unit vectors ${\vec{e}}_{k},$ k=2, …, n, we have to find the eigensystem of matrix from (43) with omitted first row and first column and all $A\left(\varepsilon^{k}\right),\;k=0,\;\ldots ,\;n-1$ again changed to the corresponding $\delta A\left(\varepsilon^{k}\right),$ i.e., the (n−1)×(n−1) matrix written in basis (51) as

(53) $$\delta M_{d}=\frac{1}{n}\left(\begin{array}{ccccc} \delta A\left(1\right) & \delta A\left(\varepsilon \right) & \delta A\left(\varepsilon^{2}\right) & \cdots & \delta A\left(\varepsilon^{n-2}\right)\\ \delta A\left(\varepsilon^{n-1}\right) & \delta A\left(1\right) & \delta A\left(\varepsilon \right) & \cdots & \delta A\left(\varepsilon^{n-3}\right)\\ \delta A\left(\varepsilon^{n-2}\right) & \delta A\left(\varepsilon^{n-1}\right) & \delta A\left(1\right) & \cdots & \delta A\left(\varepsilon^{n-4}\right)\\ \vdots & \vdots & \vdots & \ddots & \vdots \\ \delta A\left(\varepsilon^{2}\right) & \delta A\left(\varepsilon^{3}\right) & \delta A\left(\varepsilon^{4}\right) & \cdots & \delta A\left(1\right) \end{array}\right).$$

The secular equation for the matrix (53) will produce an algebraic equation of degree n−1, only one step lower than the original Equation (24). Since the matrix has δA(1) at diagonal, we can say that all eigenvalues will be shifted by δA(1)/n and then split around it as the roots of the polynomial with coefficients derived from the off-diagonal elements $\delta A\left(\varepsilon^{k}\right),$ i.e., the splitting depends on lifting the degeneracy of the set of coupling constants. A finer point to note is that, as discussed at the end of Section 2.4, the matrix (53) depends not directly on $\delta a_{i},$ but rather on components of the Fourier transform of their sequence, and thus the splitting depends not only on lifting the degeneracy, but also on how abrupt it occurs in the ordered sequence of coupling constants.

The splitting may be non-zero in linear in $\delta a_{i}$ order, for example, for the simplest possible case of n=3 and a pair of degenerate states we obtain

(54) $$\delta \lambda =\frac{1}{3}\left\{\left(\delta a_{1}+\delta a_{2}+\delta a_{3}\right)\pm \sqrt{{\left(\delta a_{1}\right)}^{2}+{\left(\delta a_{2}\right)}^{2}+{\left(\delta a_{3}\right)}^{2}-\left(\delta a_{1}\delta a_{2}+\delta a_{1}\delta a_{3}+\delta a_{2}\delta a_{3}\right)}\right\},$$

which may produce linear splitting: e.g., for $\delta a_{1}=\delta a,\;\delta a_{2}=\delta a_{3}=0$ we obtain $\delta \lambda =0,\;\frac{2}{3}\delta a.$ The corresponding eigenfunctions in this case are $\left(|\eta_{2}\rangle \pm |\eta_{3}\rangle \right)/\sqrt{2}$ that produce the familiar for three-fold symmetry (which in fact is not present here, as the nuclei are no longer assumed equivalent) combinations

(55) $$|\psi_{2}\rangle =\left(2|\xi_{1}\rangle -|\xi_{2}\rangle -|\xi_{3}\rangle \right)/\sqrt{6},\;|\psi_{3}\rangle =\left(|\xi_{2}\rangle -|\xi_{3}\rangle \right)/\sqrt{2}.$$

The case of four nuclei and three degenerate states yields a cubic equation with three real roots having their center of gravity at δA(1)/4, and even in this case the obtained explicit expressions are already too awkward to be useful and give no new insight, and increasing n pushes the problem beyond reasonable effort, while the pattern of lifting the degeneracy is given by the general result (13). 

We also note that the degenerate perturbation, similar to the non-degenerate case discussed above, does not mix functions with different projections of electron spin, and all eigenfunctions of matrix (53) remain confined to subspace spanned by basis (51) and thus include only functions $\left\{|\xi_{k}\rangle \right\},k=1,\ldots ,n$ with electron spin projection $m_{e}=+\frac{1}{2}.$ This has far-reaching consequences for the original physical problem of describing recombining spin-correlated radical pairs as set forth in [54] in the sense that for first-order perturbations the set of degenerate states remains unreachable from the initial state of the electron-singlet radical pair with all nuclear spins up. The same is true for the case of a separate radical with polarized nuclei. The more difficult to describe degenerate states simply do not participate in spin evolution. Thus, small perturbations to equivalence only change the contribution of the non-degenerate state, which is a relatively mild correction with first-order changes to energies and wavefunctions, in comparison to possible lifting of degeneracy and not small, zero order, rotation of basis. As we see, the latter does not happen here.

### 2.7. The Diagonalizing Transform

The scaling transform (38) has produced the matrix (39) that also has a circulant pattern to it. It can be seen that all its rows are generated by the single line $\left(A\left(1\right),A\left(\varepsilon \right),A\left(\varepsilon^{2}\right),\ldots ,A\left(\varepsilon^{n-1}\right)\right)$ circularly shifted one position to the right when going one row down. The eigenvectors of such a matrix are again given by consecutive powers of an *n*-th root of unity. Writing the eigenproblem for $M_{1}$ as

(56) $$M_{1}\vec{c}=\frac{1}{n}\left(\begin{array}{ccccc} A\left(1\right) & A\left(\varepsilon \right) & A\left(\varepsilon^{2}\right) & \cdots & A\left(\varepsilon^{n-1}\right)\\ A\left(\varepsilon^{n-1}\right) & A\left(1\right) & A\left(\varepsilon \right) & \cdots & A\left(\varepsilon^{n-2}\right)\\ A\left(\varepsilon^{n-2}\right) & A\left(\varepsilon^{n-1}\right) & A\left(1\right) & \cdots & A\left(\varepsilon^{n-3}\right)\\ \vdots & \vdots & \vdots & \ddots & \vdots \\ A\left(\varepsilon \right) & A\left(\varepsilon^{2}\right) & A\left(\varepsilon^{3}\right) & \cdots & A\left(1\right) \end{array}\right)\left(\begin{array}{c} c_{1}\\ c_{2}\\ c_{3}\\ \vdots \\ c_{n} \end{array}\right)=\mu \left(\begin{array}{c} c_{1}\\ c_{2}\\ c_{3}\\ \vdots \\ c_{n} \end{array}\right),$$

it can be seen that vectors of the form

(57) $${\vec{c}}_{k}={\left(1,\varepsilon^{k},\varepsilon^{2k},\ldots ,\varepsilon^{(n-1)k}\right)}^{T},\;k=0,\ldots ,n-1$$

satisfy the system (56), and the first line produces the expression for eigenvalue

(58) $$n\mu_{k}=A\left(1\right)+\varepsilon^{k}A\left(\varepsilon^{k}\right)+\varepsilon^{2k}A\left(\varepsilon^{2}\right)+\cdots +\varepsilon^{(n-1)k}A\left(\varepsilon^{\left(n-1\right)}\right),\;k=0,\;\ldots ,\;n-1.$$

Substituting expressions for $A\left(\varepsilon^{m}\right)$ (18) and rearranging terms, we obtain the sum

(59) $$n\mu_{k}=\sum_{j=1}^{n}a_{j}\left(1+\varepsilon^{k+j-1}+\varepsilon^{2(k+j-1)}+\varepsilon^{3(k+j-1)}+\cdots +\varepsilon^{\left(n-1)(k+j-1\right)}\right).$$

The expression in parentheses for each term is the sum of all n consecutive powers of one of the *n*-th roots of unity and vanishes identically unless the root is unity, so the only surviving term in the sum is for k+j−1=0(modn), or j=n+1−k(modn). We thus obtain the following eigenvalues for $M_{1}:$

(60) $$\begin{array}{cc} k=1: & \mu_{1}=a_{n};\;\\ k=2: & \mu_{2}=a_{n-1};\;\\ k=3: & \mu_{3}=a_{n-2};\;\\ k=j: & \mu_{j}=a_{n-j+1};\\ k=n: & \mu_{n}=a_{1}.\;\; \end{array}$$

The backwards-going sequence of coupling constants $a_{i}$ in (60) can be reversed by taking in (57) $\varepsilon^{n-k}={\bar{\varepsilon }}^{k}$ as the root of unity that generates the vector ${\vec{c}}_{k},$ which finally produces eigenvalues and eigenvectors of $M_{1}$ as

(61) $$\mu_{k}=a_{k},\;{\vec{c}}_{k}={\left(1,{\bar{\varepsilon }}^{\left(k-1\right)},{\bar{\varepsilon }}^{2(k-1)},\ldots ,{\bar{\varepsilon }}^{\left(n-1)(k-1\right)}\right)}^{T}/\sqrt{n},\;k=0,\ldots ,n-1,$$

and the matrix serving as the obtained diagonalizing transform for $M_{1}$ is recognized as matrix $C_{3}^{+}$ from Equation (35):

(62) $$C_{3}^{}M_{1}C_{3}^{+}=D=\mathrm{diag}\left\{a_{1},a_{,2},\ldots ,a_{n}\right\}.$$

Recalling that $M_{1}$ was itself produced from the complex zero-split matrix M via the scaling transform (38), we have

(63) $$D=C_{3}M_{1}C_{3}^{+}=C_{3}^{}C_{4}^{-1}M_{}C_{4}^{-1}C_{3}^{+},$$

where we again stress that the scaling transform with matrix $C_{4}^{-1}$ is not unitary, and so the entire transform M→D is not unitary. This matrix relation can be reversed to reconstruct the complex zero-split matrix M from diagonal matrix D as

(64) $$M=C_{4}^{}C_{3}^{+}D_{}C_{3}^{}C_{4}^{}.$$

Matrix M from (64) has equation (24) as its characteristic polynomial and gives all the nonzero roots of the original problem. It can be seen that it is constructed from a simple diagonal matrix of the original parameters $a_{i}$ by two simple transforms, (complex) rotation $C_{3}^{}$ followed by stretching $C_{4}^{}.$ The transform (64) can also be thought of as changing the characteristic polynomial in the following way:

(65) $$f_{n}(\lambda )=\sum_{k=0}^{n}A_{k}{\left(-\lambda \right)}^{n-k}\to f_{n}(\lambda )=\sum_{k=0}^{n}\left(k+1\right)A_{k}{\left(-\lambda \right)}^{n-k},$$

generating the Equation (24) from the trivial equation with $\lambda_{i}=a_{i}$.

### 2.8. The Inverse Matrix

The multiplicative decomposition (64) for the complex zero-split matrix makes it easy to obtain the inverse matrix to M by taking the inverse of both sides of (64):

(66) $$W=M^{-1}=C_{4}^{-1}C_{3}^{+}D^{-1}C_{3}^{}C_{4}^{-1},\;D^{-1}=\mathrm{diag}\left\{\frac{1}{a_{1}},\frac{1}{a_{2}},\;\ldots ,\;\frac{1}{a_{n}}\right\}.$$

The inverse matrix is thus obtained from diagonal matrix $D^{-1}$ built from reciprocal coupling constants $b_{i}=1/a_{i}$ via the same rotation $C_{3}$ and compression $C_{4}^{-1}$ instead of stretching $C_{4}.$ Introducing reciprocal complexified coupling constants $B\left(\varepsilon^{k}\right)$ similar to (18) as

(67) $$B\left(\varepsilon^{k}\right)=b_{1}+\varepsilon^{k}b_{2}+{\left(\varepsilon^{k}\right)}^{2}b_{3}+\ldots +{\left(\varepsilon^{k}\right)}^{n-1}b_{n},\;k=0,\;\ldots ,\;n-1,$$

we obtain for the inverse matrix W an expression similar to expression (37) for M:

(68) $$W=\frac{1}{n}\left(\begin{array}{ccccc} \frac{1}{n+1}B\left(1\right) & \frac{1}{\sqrt{n+1}}B\left(\varepsilon \right) & \frac{1}{\sqrt{n+1}}B\left(\varepsilon^{2}\right) & \cdots & \frac{1}{\sqrt{n+1}}B\left(\varepsilon^{n-1}\right)\\ \frac{1}{\sqrt{n+1}}B\left(\varepsilon^{n-1}\right) & B\left(1\right) & B\left(\varepsilon \right) & \cdots & B\left(\varepsilon^{n-2}\right)\\ \frac{1}{\sqrt{n+1}}B\left(\varepsilon^{n-2}\right) & B\left(\varepsilon^{n-1}\right) & B\left(1\right) & \cdots & B\left(\varepsilon^{n-3}\right)\\ \vdots & \vdots & \vdots & \ddots & \vdots \\ \frac{1}{\sqrt{n+1}}B\left(\varepsilon \right) & B\left(\varepsilon^{2}\right) & B\left(\varepsilon^{3}\right) & \cdots & B\left(1\right) \end{array}\right)$$

that differs from (37) only by changing all $A\left(\varepsilon^{k}\right)$ to their reciprocal counterparts $B\left(\varepsilon^{k}\right)$ and all multiplicative prefactors $n+1,\sqrt{n+1}$ also to their reciprocals.

This reciprocity-type connection between the matrix and its inverse continues to their spectral properties. If a non-degenerate matrix A has the set $\left\{\lambda_{i}\right\}$ as its spectrum, its inverse $A^{-1}$ has spectrum $\left\{\mu_{i}=\lambda_{i}^{-1}\right\}$ with the same ordering of eigenvalues, and is diagonalized by the same transform. Indeed [101], if

(69) $$C^{+}A_{}C=\mathrm{diag}\left\{\lambda_{1},\;\ldots ,\;\lambda_{n}\right\}=\tilde{A},\;A=C\tilde{A}C^{+},$$

take

(70) $$\tilde{B}=\mathrm{diag}\left\{\frac{1}{\lambda_{1}},\;\ldots ,\;\frac{1}{\lambda_{n}}\right\}.$$

Apparently,

(71) $$\tilde{A}\tilde{B}=\tilde{B}\tilde{A}=\overset{⌢}{1},$$

So, we can write

(72) $$\overset{⌢}{1}=C\overset{⌢}{1}C^{+}=C\tilde{A}C^{+}C\tilde{B}C^{+}=C\tilde{B}C^{+}C\tilde{A}C^{+}=AC\tilde{B}C^{+}=C\tilde{B}C^{+}A,$$

and from uniqueness of the inverse matrix, we get

(73) $$A^{-1}=C\tilde{B}C^{+},\;C^{+}A^{-1}C=\tilde{B}=\mathrm{diag}\left\{\frac{1}{\lambda_{1}},\;\ldots ,\;\frac{1}{\lambda_{1}}\right\},$$

with the same transforming matrix C, i.e., the same set of eigenvectors taken in the same order, and with reciprocal eigenvalues taken in the same order.

Finally, the reciprocal connection continues to the characteristic polynomial (24). Taking advantage of the fact that λ=0 is not a root, dividing Equation (24) by ${\left(-\lambda \right)}^{n}A_{n}(n+1),$ and introducing $\mu =\lambda^{-1},$ we obtain the following pair of equations:

(74) $$\begin{array}{l} f_{n}(\lambda )={\left(-\lambda \right)}^{n}+2A_{1}{\left(-\lambda \right)}^{n-1}+3A_{2}{\left(-\lambda \right)}^{n-2}+4A_{3}{\left(-\lambda \right)}^{n-3}+\cdots +\left(n+1\right)A_{n},\\ {\tilde{f}}_{n}(\mu )={\left(-\mu \right)}^{n}+\frac{n}{n+1}B_{1}{\left(-\mu \right)}^{n-1}+\frac{n-1}{n+1}B_{2}{\left(-\mu \right)}^{n-2}+\frac{n-2}{n+1}B_{3}{\left(-\mu \right)}^{n-3}+\cdots +\frac{1}{n+1}B_{n}, \end{array}$$

where we naturally obtain the reciprocal counterpart of definitions (7)

(75) $$B_{1}=\frac{A_{n-1}}{A_{n}}=\sum_{i=1}^{n}\frac{1}{a_{i}}=\sum_{i=1}^{n}b_{i},\;B_{2}=\frac{A_{n-2}}{A_{n}}=\sum_{\begin{array}{l} i,j=1\\ \;i<j \end{array}}^{n}\frac{1}{a_{i}a_{j}}=\sum_{\begin{array}{l} i,j=1\\ \;i<j \end{array}}^{n}b_{i}b_{j},\;\mathrm{etc}.$$

The roots of the two equations in (74) are related as $\mu_{i}=\lambda_{i}^{-1}$.

The essence of last sections can be more usefully summarized if we treat the coupling constants $a_{i}$ simply as numbers rather than dimensional physical parameters, so that $a_{i}$ and $a_{i}^{-1}$ can coexist in the same space and be plotted in the same axes. Then we can take two diagonal matrices, written in the same basis and having either $a_{i}$ or $a_{i}^{-1}$ at diagonal, stretch one matrix by a factor of $\sqrt{n+1}$ and compress the other one by the same factor along the same direction, the spatial diagonal ${\vec{e}}_{d}={\left(1,\;1,\;\ldots ,\;1\right)}^{T}/\sqrt{n},$ and obtain the matrix that produces the sought nonzero roots for the original physical problem and its inverse, with their eigenvalues related reciprocally. This duality and the possibility to obtain simultaneously both the sought roots and their inverses will prove very useful for geometrization that we introduce in the following section.

### 2.9. The Geometrization

A geometric image of a real symmetric positive definite n×n matrix is an n-dimensional hyper-ellipsoid in real n-space obtained by constructing a quadratic form from the matrix. For simplicity we shall omit “hyper” and refer to them simply as ellipsoids. For a diagonal matrix $D=\mathrm{diag}\left\{a_{1},\;a_{,2},\;\ldots ,\;a_{n}\right\}$ we construct the form

(76) $$1={\vec{x}}^{T}D_{}\vec{x}=\sum_{i}a_{i}x_{i}^{2}=\sum_{i}{\left(\frac{x_{i}}{p_{i}}\right)}^{2},\;p_{i}=\frac{1}{\sqrt{a_{i}}}$$

and obtain an ellipsoid with semiaxes given by inverse square roots of the diagonal elements, aligned with the Cartesian axes of the n-space. For a general real positive definite matrix, we obtain a similar ellipsoid, but rotated and with semiaxes built from eigenvalues as $p_{i}=1/\sqrt{\lambda_{i}}.$ The same is true for a complex Hermitian positive definite matrix, although it may be not straightforward to visualize it. Still some unitary transform, i.e., pure rotation, converts it into a real diagonal matrix without modifying its spectrum, and the ellipsoid visualization remains valid.

Therefore, the transforms that simultaneously reconstruct the matrices M containing all the nonzero roots of the original problem (64) and its inverse W (66) from diagonal matrices $D=\mathrm{diag}\left\{a_{1},\;a_{2},\;\ldots ,\;a_{n}\right\}$ and $D^{-1}=\mathrm{diag}\left\{1/a_{1},\;1/a_{2},\;\ldots ,\;1/a_{n}\right\}$ correspond to scaling the ellipsoids with semiaxes $1/\sqrt{a_{i}}$ and $\sqrt{a_{i}},$ respectively, to produce different ellipsoids with semiaxes $1/\sqrt{\lambda_{i}}$ and $\sqrt{\lambda_{i}},$ respectively. While formally we have to stretch/compress a complex Hermitian matrix rather than real symmetric one, the transform actually proceeds along the only explicitly real basis vector ${\vec{e}}_{d}={\left(1,\;1,\;\ldots ,\;1\right)}^{T}/\sqrt{n}$ and does not change anything in its complementary (n−1)-dimensional space spanned by the complex basis vectors. If needed, an additional unitary transform can be performed in this complementary space to obtain a real basis and a real symmetric matrix with the same spectrum, and thus explicitly transform an ellipsoid to an ellipsoid. This is the “shuffling back” transform (19) that gave us the real symmetric matrix out of the complex zero-split one, but in doing so ruined the symmetry that was later used to diagonalize the matrix. We now see that this additional transform, confined to the complementary space, is immaterial, and the net result is geometrically stretching/compressing an *n*-dimensional ellipsoid by a factor of $\sqrt{n+1}$ along the direction ${\vec{e}}_{d}={\left(1,\;1,\;\ldots ,\;1\right)}^{T}/\sqrt{n}$.

This is almost what we strived to achieve, the only nuisance being the recurring reciprocity that interferes with intuition. It is natural to work with matrix M corresponding to the original problem, and think in terms of its representing ellipsoid. However, the semiaxes of the starting ellipsoid for M are the *inverse* square roots of the corresponding parameters $a_{i},$ the sought roots $\lambda_{i}$ are the *inverse* squared semiaxes of the resulting ellipsoid, and the stretching of matrix is geometrically the compressing of its representing ellipsoid. However, all this is taken care of if we consider the dual transform of the inverse matrix $D^{-1}:$ the semiaxes of the starting ellipsoid are now $\sqrt{a_{i}},$ the squared semiaxes of the resulting ellipsoid are now $\lambda_{i},$ and the compressing of the matrix implied by transform (66) is the spatial stretching of the ellipsoid.

We have thus constructed a simple geometric visualization procedure for the roots of the zero-split matrix (64): given n spin-12 nuclei with arbitrary positive coupling constants $a_{i},$ take an *n*-dimensional ellipsoid centered at the origin of the reference frame and having semiaxes $\sqrt{a_{i}}$ aligned with the *n* Cartesian axes, stretch it by a factor of $\sqrt{n+1}$ along the spatial diagonal ${\vec{e}}_{d}={\left(1,\;1,\;\ldots ,\;1\right)}^{T}/\sqrt{n},$ and the semiaxes of the obtained new ellipsoid $q_{i}$ produce the n sought positive roots as $\lambda_{i}=q_{i}^{2},\;i=1,\;\ldots ,\;n.$ Augment this set with additional zero root $\lambda_{0}=q_{0}^{2}=0,$ and obtain the n+1 energy levels of the penultimate Hamiltonian block as

(77) $$E_{k}=-\frac{1}{2}q_{k}^{2}+\frac{1}{4}\sum_{i=1}^{n}a_{i},\;k=0,\;\ldots ,\;n.$$

**Figure 2:**
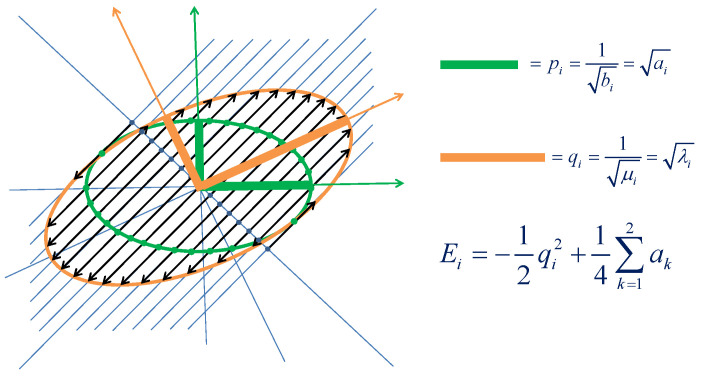
Stretching the representing ellipse for a two-nuclei system with $a_{1}=25$ and $a_{2}=9$ relative units, corresponding to semiaxes of 5 and 3 r.u., along the direction ${\left(1,1\right)}^{T}/\sqrt{2}$ by a factor of $\sqrt{3}$ to obtain the roots as the squared semiaxes of the transformed ellipse. The figure shows the initial and resulting ellipses with their semiaxes as bold sticks, the stretch lines along the ${\left(1,1\right)}^{T}/\sqrt{2}$ direction and the reference line normal to them, the old and new Cartesian axes for the ellipses, and the correspondence of the points at the cross-section of the initial ellipse with the stretch lines and their images at the final ellipse that are a factor of $\sqrt{3}$ farther away from the reference line along the stretch lines.

Figure 2 shows graphically how stretching a 2D ellipse by a factor of $\sqrt{3}$ along the ${\left(1,1\right)}^{T}/\sqrt{2}$ direction produces a transformed and rotated ellipse with larger semiaxes corresponding to the sought roots for a system with two nuclei, illustrating the solutions $\lambda_{1,2}=\left(a_{1}+a_{2}\right)\pm \sqrt{a_{1}^{2}+a_{2}^{2}-a_{1}a_{2}}$ to equation $\lambda^{2}-2\left(a_{1}+a_{2}\right)+3a_{1}a_{2}=0$.

**Figure 3:**
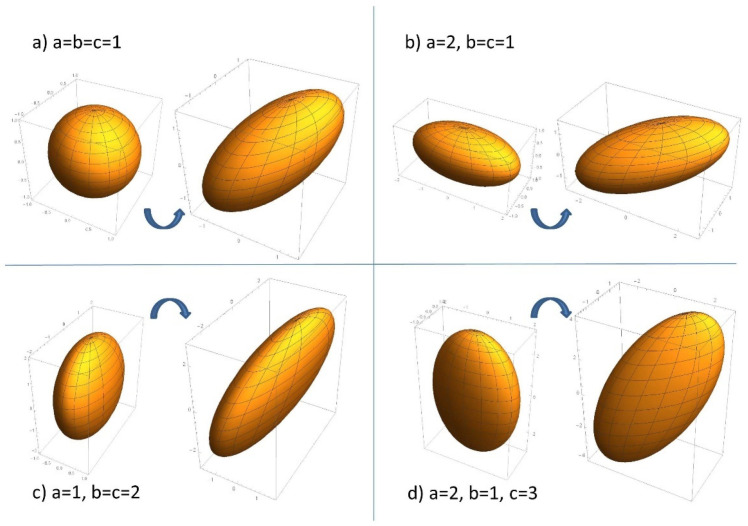
The transform for several representative cases of a three-nuclei system: stretching a 3D ellipsoid a factor of 2 along the spatial diagonal ${\left(1,1,1\right)}^{T}/\sqrt{3}.$ Legends in the panels indicate semiaxes of the starting ellipsoid in r.u. that are equal to square roots of the original hyperfine coupling constants $a_{i},$ while squared semiaxes $q_{i}$ of the stretched ellipsoids give the sought energies as $E_{i}=-\frac{1}{2}q_{i}^{2}+\frac{1}{4}\sum_{k=1}^{3}a_{k},\;i=0,\;\ldots ,\;3,\;q_{0}=0$.

Figure 3 shows the operation of the developed transform for a three-nuclei system, when the representing ellipsoid is a conventional 3D ellipsoid that can still be relatively easily drawn and visualized. The figure shows four panels for the representative cases of the starting ellipsoid having the shape of a sphere (all three semiaxes are equal, a=b=c), oblong ellipsoid of revolution (one longer semiaxis and two equal ones, a>b=c), oblate ellipsoid of revolution (one shorter semiaxis and two equal ones, a=b<c), and a general ellipsoid (all three semiaxes are different). The starting ellipsoid with its semiaxes aligned with Cartesian axes is stretched by a factor of 2 along the spatial diagonal ${\left(1,1,1\right)}^{T}/\sqrt{3}.$ The images were generated in Mathematica by parametrizing the initial ellipsoid as

(78) x=asinθcosϕ, y=bsinθsinϕ, z=ccosθ, θ∈[0, π], ϕ∈[0, 2π],

applying the transform to the radius-vector as

(79) $$\left(\begin{array}{c} x^{\prime }\\ y^{\prime }\\ z^{\prime } \end{array}\right)={\vec{r}}^{\prime }=\hat{R}\hat{S}{\hat{R}}^{+}\vec{r}=\left(\begin{array}{ccc} \frac{1}{\sqrt{3}} & 0 & -\frac{2}{\sqrt{6}}\\ \frac{1}{\sqrt{3}} & \frac{1}{\sqrt{2}} & \frac{1}{\sqrt{6}}\\ \frac{1}{\sqrt{3}} & -\frac{1}{\sqrt{2}} & \frac{1}{\sqrt{6}} \end{array}\right)\left(\begin{array}{ccc} 2 & 0 & 0\\ 0 & 1 & 0\\ 0 & 0 & 1 \end{array}\right)\left(\begin{array}{ccc} \frac{1}{\sqrt{3}} & \frac{1}{\sqrt{3}} & \frac{1}{\sqrt{3}}\\ 0 & \frac{1}{\sqrt{2}} & -\frac{1}{\sqrt{2}}\\ -\frac{2}{\sqrt{6}} & \frac{1}{\sqrt{6}} & \frac{1}{\sqrt{6}} \end{array}\right)\left(\begin{array}{c} x\\ y\\ z \end{array}\right)=\hat{P}\vec{r},$$

and using the ParametricPlot3D function to draw the initial and the transformed ellipsoids shown in the figure.

When building the transform (79) to generate the figures we took advantage of the freedom to choose the basis vectors in the orthogonal complement to the stretching direction mentioned above, and used a pair of real vectors instead of complex ones generated by cubic roots of 1 to embed the figure in ordinary 3D space. A careful study of Figure 2 and Figure 3 illustrates the general spectral bounds of (12) and (13): the stretching either leaves some semiaxes unchanged for the degenerate case of the ellipsoids of revolution, of increases them for differing semiaxes, but so as to not step over the next larger one, and the longest semiaxis of the initial ellipsoid always becomes longer.

A couple of simple examples can also be provided for systems of higher dimensions. The first one would be n equivalent nuclei with the same coupling constant a. The ellipsoid then turns into an *n*-dimensional sphere of radius $\sqrt{a},$ and stretching it in any direction by a factor of $\sqrt{n+1}$ produces an ellipsoid of revolution with one semiaxis equal to $\sqrt{(n+1)a}$ and remaining n−1 semiaxes unchanged at $\sqrt{a},$ which gives the solution (29).

The second example would be n−1 equivalent nuclei with the coupling constants $a_{1}=\cdots =a_{n-1}=a$ and one nucleus with a larger coupling $a_{n}>a.$ The starting ellipsoid for this configuration is an ellipsoid of revolution with one long semiaxis $\sqrt{a_{n}}$ and the remaining n−1 equal semiaxes $\sqrt{a},$ which we have to stretch along direction ${\vec{e}}_{d}={\left(1,\;1,\;\ldots ,\;1\right)}^{T}/\sqrt{n}.$ The stretching will be confined to plane $\sigma_{dn}$ spanning vectors ${\vec{e}}_{d}$ and ${\vec{e}}_{n}={\left(0,\;0,\;\ldots ,\;0,\;1\right)}^{T},$ with the latter corresponding to the long semiaxis. The stretched ellipsoid will have n−2 semiaxes normal to $\sigma_{dn}$ staying at $\sqrt{a}$ that produce the (n−2)-degenerate root $\lambda_{1}^{}=a.$ The restriction of the ellipsoid to plane $\sigma_{dn}$ is an ellipse with semiaxes $\sqrt{a}$ and $\sqrt{a_{n}}$ that we now have to stretch by a factor of $\sqrt{n+1}$ along the direction with angle γ to axis ${\vec{e}}_{n}={\left(0,\;0,\;\ldots ,\;0,\;1\right)}^{T}$ given by

(80) $$\cos\gamma ={\vec{e}}_{n}\cdot \;{\vec{e}}_{n}=\frac{1}{\sqrt{n}},\;\sin\gamma =\sqrt{\frac{n-1}{n}}.$$

To find the semiaxes of the stretched ellipse analytically, we introduce rotation in plane $\sigma_{dn}$ as

(81) $$\left(\begin{array}{c} x\\ y \end{array}\right)\to \left(\begin{array}{c} x^{\prime }\\ y^{\prime } \end{array}\right)=\left(\begin{array}{cc} \cos\gamma & \sin\gamma \\ -\sin\gamma & \cos\gamma \end{array}\right)\left(\begin{array}{c} x\\ y \end{array}\right)=\left(\begin{array}{c} \frac{1}{\sqrt{n}}x+\sqrt{\frac{n-1}{n}}y\\ -\sqrt{\frac{n-1}{n}}x+\frac{1}{\sqrt{n}}y \end{array}\right),$$

stretch $x^{\prime }$ by a factor of $\sqrt{n+1}$ to obtain

(82) $$\left(\begin{array}{c} x^{\prime \prime }\\ y^{\prime \prime } \end{array}\right)=\left(\begin{array}{c} \sqrt{n+1}x^{\prime }\\ y^{\prime } \end{array}\right)=\left(\begin{array}{c} \sqrt{\frac{n+1}{n}}x+\sqrt{\frac{n^{2}-1}{n}}y\\ -\sqrt{\frac{n-1}{n}}x+\frac{1}{\sqrt{n}}y \end{array}\right),$$

construct the distance to origin

(83) $$r^{2}={\left(x^{\prime \prime }\right)}^{2}+{\left(y^{\prime \prime }\right)}^{2}=2x^{2}+ny^{2}+2\sqrt{n-1}xy,$$

and search for its extremum. To do this we parametrize the point at the original ellipse via its angle

(84) $$x=p\cos\phi ,\;y=q\sin\phi ,\;p=\sqrt{a_{n}},\;q=\sqrt{a},$$

and from (83) obtain

(85) $$r^{2}=2p^{2}\cos^{2}\phi +nq^{2}\sin^{2}\phi +2pq\sqrt{n-1}\sin\phi \cos\phi ,$$

which has extremum at

(86) $$\tan2\phi_{0}=\frac{2pq\sqrt{n-1}}{2p^{2}-nq^{2}}$$

given by

(87) $$r^{2}|{}_{\phi_{0}}=p^{2}+\frac{n}{2}q^{2}+\frac{1}{2}\sqrt{4p^{4}+n^{2}q^{4}4p^{2}q^{2}}=a_{n}+\frac{n}{2}a+\sqrt{a_{n}^{2}+{\left(\frac{n}{2}\right)}^{2}a^{2}-aa_{n}}=\lambda_{2}.$$

The remaining distinct root $\lambda_{3}$ can be obtained from the scaling of determinants of the matrices M and D. Taking determinants at both sides of (64) we obtain:

(88) $$\prod_{i}\lambda_{i}=a^{n-2}\lambda_{2}^{}\lambda_{3}=\left|M\right|=\left|C_{4}^{}C_{3}^{+}D_{}^{}C_{3}^{}C_{4}^{}\right|=\left(n+1\right)\prod_{i}a_{i}=\left(n+1\right)a^{n-1}a_{n},$$

and $\lambda_{3}$ is found to be

(89) $$\lambda_{3}=a_{n}+\frac{n}{2}a-\sqrt{a_{n}^{2}+{\left(\frac{n}{2}\right)}^{2}a^{2}-aa_{n}}.$$

A more direct way to obtain the three roots $\lambda_{1,2,3}$ in this particular case is to use only the general properties of roots (13) and the expression for determinant and trace of matrix M (40). We know that for n−1 equivalent nuclei out of n we must have an (n−2)-degenerate root $\lambda_{1}=a$ and two other unknown roots $\lambda_{2},\;\lambda_{3},$ and we can write two simple equations for the trace and determinant of the matrix as

(90) $$\begin{array}{l} \sum_{i}\lambda_{i}=\left(n-2\right)a+\lambda_{2}+\lambda_{3}=2A_{1}=2\left\{\left(n-1\right)a+a_{n}\right\},\\ \prod_{i}\lambda_{i}=a^{n-2}\lambda_{2}^{}\lambda_{3}=\left(n+1\right)A_{n}=\left(n+1\right)a^{n-1}a_{n}, \end{array}$$

from which we have

(91) $$\begin{array}{l} \lambda_{2}+\lambda_{3}=na+2a_{n},\;\lambda_{2}\lambda_{3}=\left(n+1\right)aa_{n},\\ \lambda^{2}-\lambda \left(na+2a_{n}\right)+\left(n+1\right)aa_{n}=0,\\ \lambda_{2,3}=a_{n}+\frac{n}{2}a\pm \sqrt{a_{n}^{2}+{\left(\frac{n}{2}\right)}^{2}a^{2}-aa_{n}}, \end{array}$$

which coincides with (87), (89).

In these simples cases the solutions provided by the geometric approach are certainly more complex than the straightforward algebraic solutions to quadratic equations. For even slightly more complex systems both approaches fail to produce closed-form solutions. This is that heaping up complexity behind the simple to imagine or draw things referred to in the Introduction. However, while for the algebraic approach this is a plain dead end, the geometric visualization still remains valid, and it is earnestly believed, useful.

### 2.10. Variational Derivation of Characteristic Equation 

The characteristic Equation (9) can be obtained from the suggested transform variationally for a general situation of n arbitrary coupling constants $a_{1},\;\ldots ,\;a_{n}.$ Take an *n*-dimensional ellipsoid with semiaxes $\sqrt{a_{i}}$ and arbitrarily select a point at it having radius-vector ${\vec{r}}_{0}.$ The reference plane for the stretching is the plane orthogonal to unit vector ${\vec{e}}_{d}={\left(1,\;1,\;\ldots ,\;1\right)}^{T}/\sqrt{n},$ the distance from our point to this plane is equal to $d={\vec{e}}_{d}\cdot \;{\vec{r}}_{0},$ the stretching does not change the component of ${\vec{r}}_{0}$ in the reference plane and scales the distance d by a factor of $\sqrt{n+1},$ i.e., the overall transform can be expressed as

(92) $${\vec{r}}_{0}\to \vec{r}={\vec{r}}_{0}+h\left({\vec{e}}_{d}\cdot \;{\vec{r}}_{0}\right){\vec{e}}_{d},\;h=\sqrt{n+1}-1.$$

The semiaxes of the ellipsoid are extremal distances from the origin, so build a distance-related functional by taking the squared distance to origin $r^{2}$ and apply the constraint that the original point ${\vec{r}}_{0}$ is at the ellipsoid, and search for its extremum:

(93) $$F\left({\vec{r}}_{0}\right)=\vec{r}\left({\vec{r}}_{0}\right)\;\cdot \;\vec{r}\left({\vec{r}}_{0}\right)-\lambda \left(\sum_{k=1}^{n}{\left(\frac{r_{0k}}{\sqrt{a_{k}}}\right)}^{2}-1\right)\to \max,$$

where λ is the Lagrange multiplier and the sum runs over all Cartesian coordinates of the vector ${\vec{r}}_{0}.$ Building the functional (93), taking partial derivatives with respect to $r_{0k}$ and setting them equal to zero produces the system of equations

(94) $$r_{0k}\left(1-\frac{\lambda }{a_{k}}\right)=-\sqrt{n}\left({\vec{e}}_{d}\cdot \;{\vec{r}}_{0}\right)=const(k),\;k=1,\;\ldots ,\;n,$$

where ${\vec{e}}_{d}\cdot \;{\vec{r}}_{0}$ is unknown, but the same for each equation in the system (94). To eliminate it, use the coordinates $r_{0k}$ from (94) to construct ${\vec{e}}_{d}\cdot \;{\vec{r}}_{0}$ and obtain

(95) $${\vec{e}}_{d}\cdot \;{\vec{r}}_{0}=-\sum_{k=1}^{n}\frac{{\vec{e}}_{d}\cdot \;{\vec{r}}_{0}}{1-\frac{\lambda }{a_{k}}}.$$

Since (95) must hold for arbitrary ${\vec{r}}_{0}$ at the ellipsoid and ${\vec{e}}_{d}$ is fixed, we can cancel ${\vec{e}}_{d}\cdot \;{\vec{r}}_{0}$ at both sides to finally obtain

(96) $$1+\sum_{k=1}^{n}\frac{a_{k}}{a_{k}-\lambda }=0,$$

which is recognized as Equation (9), one of the forms of the original secular equation that was used as the starting point of this work. Thus, the suggested transform produces the original secular equation via a variational procedure, and the eigenvalue appears as the Lagrange multiplier in the varied functional, as is common in variational formulation of physical problems.

## 3. Conclusions

Although the suggested visualization procedure cannot yield analytic expressions for the eigenvalues beyond simple cases that can be directly calculated, it does remain valid for an arbitrary complex system, when the calculations have long become impossible and the only option is finding the eigenvalues numerically anyway, while the complexity of visualization does not increase. It is believed that such thinking of Hamiltonians in terms of geometric shapes can be useful in supporting the intuition and providing insights for complex systems to complement the usually inevitable numeric calculations. 

It would also be useful to track the entire process of transformations that have led from the starting matrix for the Hamiltonian block to the n-dimensional ellipsoid defined simply by the coupling constants $a_{i}.$ Finding eigenvalues of a matrix can be viewed as going from the initial basis to the basis of its eigenvectors, and, although this can be, and numerically almost always is, done directly in one step, the ability to decompose it into a sequence of more comprehendible and visualizable steps by going through several intermediate bases can provide new insights about the general properties of the system. In our case the initial matrix was first shifted, uniformly scaled and changed in sign to obtain the Laplacian-like matrix (5) that is guaranteed to have one zero root and all remaining roots positive, which can be seen as a hyper-paraboloid, a higher-dimensional generalization of the shape of a wine glass. After such conditioning of the initial matrix to generate the starting representation for geometrization, all subsequent steps can be viewed as manipulations with geometric shapes. First the zero splitting transform was applied to obtain a smaller positive definite matrix (37), which can be seen as taking a cross-section of the hyperboloid normal to its axis, direction ${\left(1,\;1,\;\ldots ,\;1\right)}_{n+1}^{T}$ in (n+1)-dimensional space, and obtaining a hyper-ellipsoid one step lower in dimension, similar to looking at the glass from the top to see a circular pattern. Then a stretching transform along direction ${\left(1,\;1,\;\ldots ,\;1\right)}_{n}^{T}$ in the new space (38) produced a simpler hyper-ellipsoid, which could be rotated (62) to align it with Cartesian axes of its space, i.e., to diagonalize the corresponding matrix. This sequence of transforms is not unitary all way through and it does modify the spectrum, as it involves a non-unitary stretching transform in the middle, but it still can be seen as two legs of unitary rotations connected by an easily visualizable non-unitary bridge of the stretch, in which all the algebraic complexity of the problem is condensed. Such “click algebra” of going in simple steps between consecutive bases is believed to be useful for analyzing matrix-related problems common in science. 

## Figures and Tables

**Table 1:** Energies E and corresponding roots λ for subspace $K=K_{\max}-1$.

$I_{\Sigma }$	J	$E_{}$	λ	Multiplicity
$\frac{n}{2}$	$\frac{n+1}{2}$	$\frac{na}{4}$	0	1
$\frac{n}{2}$	$\frac{n-1}{2}$	$-\frac{a}{2}\left(\frac{n}{2}+1\right)$	(n+1)a	1
$\frac{n}{2}-1$	$\frac{n-1}{2}$	$\frac{a}{2}\left(\frac{n}{2}-1\right)$	a	n−1
